# Foraging for water by MIZ1-mediated antagonism between root gravitropism and hydrotropism

**DOI:** 10.1073/pnas.2427315122

**Published:** 2025-05-15

**Authors:** Yuzhou Zhang, Zhulatai Bao, Adrijana Smoljan, Yifan Liu, Huihui Wang, Jiří Friml

**Affiliations:** ^a^Department of Biochemistry, College of Life Sciences, Northwest A&F University, Yangling, Shaanxi 712100, China; ^b^State Key Laboratory for Crop Stress Resistance and High-Efficiency Production, Northwest A&F University, Yangling, Shaanxi 712100, China; ^c^Institute of Future Agriculture, Northwest A&F University, Yangling, Shaanxi 712100, China; ^d^Institute of Science and Technology Austria, Klosterneuburg 3400, Austria

**Keywords:** root hydrotropism, root gravitropism, drought avoidance, auxin transport, root system architecture

## Abstract

Root system integrates multiple environmental cues, chiefly gravity and soil humidity, to anchor plants in soil and forage for water. While the mechanism of auxin-mediated root gravitropism is comparably well-understood, the root’s capability to grow toward moist soil for water uptake and drought avoidance, termed root hydrotropism, remains largely mysterious. Here, we provide key insights into the mechanism of hydrotropic growth and assign a role to the master regulator of hydrotropism, MIZU-KUSSEI 1 (MIZ1). We show that efficient hydrotropism requires the attenuation of antagonistically acting gravitropism, which is inhibited under drought conditions. Drought stress interferes with subcellular trafficking and the lateral mobility of PIN auxin transporters, which are polarly localized at the root cell plasma membranes. This leads to defects in PIN2 polarity and gravity-induced polarization of PIN3, ultimately inhibiting gravity-induced auxin redistribution and root bending. The *miz1* mutant is defective in all these regulations, and in support of MIZ1’s action on PINs, *pin* mutations rescue the hydrotropic defects in the *miz1* mutant. These observations identify a mechanism for how drought via MIZ1 attenuates gravitropism to promote root hydrotropism for efficient water foraging under drought conditions.

## Significance

Root hydrotropism is critical for a plant’s ability to seek water. Despite its crucial role in adapting to drought, the underlying mechanisms remain elusive. We reveal that the master hydrotropism regulator, MIZU-KUSSEI 1 (MIZ1), inhibits gravitropism to promote hydrotropic root bending. MIZ1 achieves this by dynamically tuning the polarity of PIN auxin transporters at the plasma membrane in response to fluctuations in environmental water potential. This mechanism allows plants to adjust the PIN-mediated auxin redistribution rate along the root longitudinal axis, promoting hydrotropic growth and shaping root system architecture for optimal water uptake. This MIZ1-PIN regulatory module translates environmental water availability into auxin signaling, guiding the “hidden part” of plants to locate water in the underground environment.

Terrestrial plants are sessile organisms. To acclimate rapidly to their surrounding environment, they have evolved exceptional developmental plasticity. Fascinating examples are tropisms, the directed growth responses to environmental stimuli such as gravity, light, nutrients, and water in the soil (1, 2). Root gravitropism, one of the most studied tropisms (3–6), allows plant roots to grow downward along the gravity vector, facilitating root anchoring in the soil and enabling the acquisition of nutrients and water. Nevertheless, gravitropism alone is insufficient for plants to acquire water efficiently, as water distribution in the soil is often heterogeneous. Therefore, to effectively forage for water, plant roots have evolved a unique capability to sense moisture gradients in the soil and then bend toward regions of higher water potential (Wp), which is termed hydrotropism (7, 8). Hydrotropism-driven water acquisition helps plants overcome the detrimental effects of drought stress (9).

The molecular mechanism of root gravitropism has been extensively studied (4–6). Mechanistically, gravity sensing occurs through the sedimentation of starch-filled amyloplasts within the root apex (3), which leads to the asymmetrical distribution of auxin between the lower and upper sides of the root. This process relies on polar auxin transport mediated by PIN auxin efflux transporters, and the asymmetric auxin redistribution results in differential growth rates between the upper and lower sides of the root, thus enabling roots to bend downward (5, 10).

In addition to gravitropism, other types of plant tropic growth, including phototropism (11, 12), thigmotropism (13), and halotropism (14, 15), presumably require the PIN-mediated asymmetric auxin distribution. However, limited observations suggest that root hydrotropic growth largely relies on the phytohormones cytokinin and abscisic acid (ABA) rather than auxin (15–19), as indicated by the following observations: i) Auxin-responsive reporters do not show asymmetry during root hydrotropic bending (18, 19); instead, it requires ABA signaling and the asymmetric distribution of cytokinin mediated by MIZU-KUSSEI 1 (MIZ1) protein (17, 20); ii) The hydrotropism-defective *miz1* mutant shows normal root gravitropism (8), suggesting distinct mechanisms for these two responses; iii) Pharmacological interference with auxin transport fails to inhibit root hydrotropism but almost completely prevented other types of plant tropic responses, such as gravitropism and phototropism (19, 21).

Nonetheless, some experiments support the involvement of auxin in root hydrotropism. For example, interference with auxin signaling enhances root hydrotropism (22), and reactive oxygen species and calcium (Ca^2+^), which are linked to auxin signaling, also function in root hydrotropism (23, 24). Moreover, recent studies indicated that root gravitropism and hydrotropism act antagonistically (25, 26), but it remains entirely unclear how the responses to these two distinct environmental stimuli, gravity and water, are linked. Due to these disparate views, the potential role of auxin in root hydrotropism remains elusive, as does the mechanism of hydrotropism itself.

This study provides key insights into how roots forage for water. We revealed that root hydrotropism requires the attenuation of gravitropism, which is induced by drought stress and mediated by the master hydrotropism regulator MIZ1. Furthermore, we establish the cellular mechanism by which MIZ1 affects gravity-induced, PIN transporter–mediated auxin redistribution to attenuate gravitropism, thereby promoting root hydrotropism.

## Results

### MIZ1 Regulates Root Hydrotropism by Antagonizing the Root Gravitropism.

**Fig. 1.**
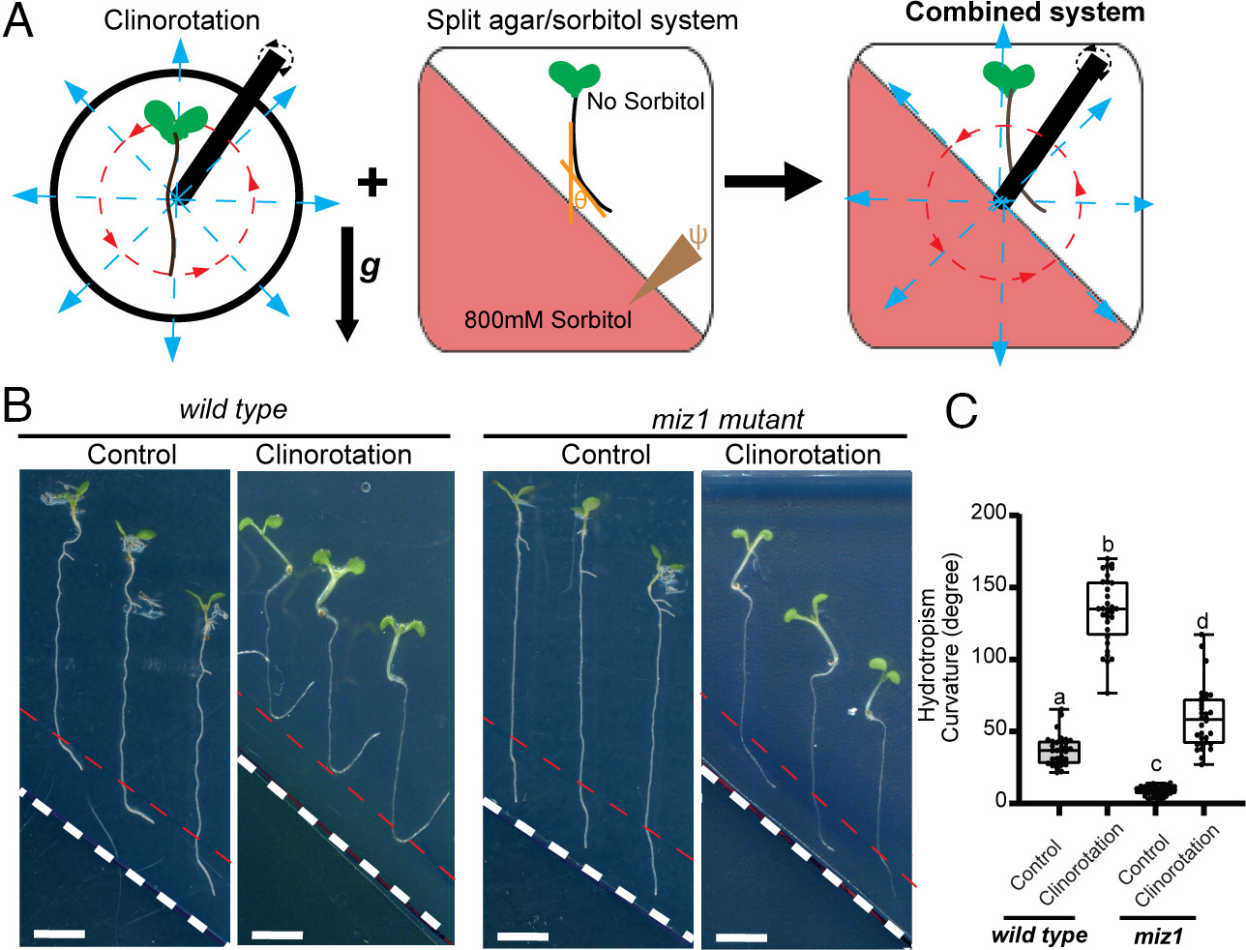
Interaction of root gravitropism and MIZ1-dependent hydrotropism. (*A*) A modified clinostat device combined with a split agar/sorbitol system was used to explore root hydrotropic growth in a zero- or microgravity environment. (*B*) The effects of clinorotation on the root hydrotropic response of *Arabidopsis* WT and *miz1* mutant. (Scale bars, 5 mm.) The white dotted lines indicate the boundary between the agar media with 800 mM sorbitol (*Bottom Left*) and without sorbitol (*Top Right*). The red dotted lines mark the initial position of *Arabidopsis* root tip before it grew for 24 h in the split agar/sorbitol system. (*C*) Quantification of the root hydrotropic bending angles for each group (Three biological replicates for each group, *n* ≥ 10 roots for each replicate) in (*B*). Each circle represents the measurement of an individual root. Boxplots span the first to the third quartiles of the data. Whiskers indicate the minimum and maximum values. A line in the box represents the mean. Significant differences were determined by one-way ANOVA followed by Tukey’s multiple comparisons test, with a significance level of *P* < 0.001.

A clinostat is commonly used to simulate plant growth in a zero- or microgravity environment by randomizing the gravity vector, which nullifies plant gravitropic response (27). Meanwhile, the split-agar/sorbitol system, which generates a Wp gradient in the agar medium (7), is widely used to study root hydrotropic growth. Here, we designed a device that combines both methods by placing the split-agar/sorbitol system in a clinostat to investigate root hydrotropic growth with a limited gravitropic response (Fig. 1*A*). Our results showed that clinorotation substantially increases the *Arabidopsis* root hydrotropic bending angle (Fig. 1*B*), suggesting that inhibition of root gravitropism enhances root hydrotropism.

*MIZ1* has been identified as one of the critical components of root hydrotropism (8). It has been reported that MIZ1 mediates root hydrotropism, thus improving *Arabidopsis* performance under drought conditions (9). To determine whether MIZ1 is involved in the antagonistic interaction between root hydrotropic and gravitropic growth, we analyzed the hydrotropic phenotype of the *miz1* mutant in the clinostat. After circumventing the influence of gravity on root growth by clinorotation, the defective root hydrotropism of the *miz1* mutant was partially rescued, with a significantly increased root bending toward the high Wp side in the split-agar/sorbitol system (Fig. 1 *B* and *C*).

Together, these findings suggest that root gravitropism antagonizes hydrotropism and that the role of MIZ1 in root hydrotropism involves attenuating root gravitropism.

### Sorbitol Treatment Attenuates Auxin-Mediated Root Gravitropism via MIZ1.

**Fig. 2.**
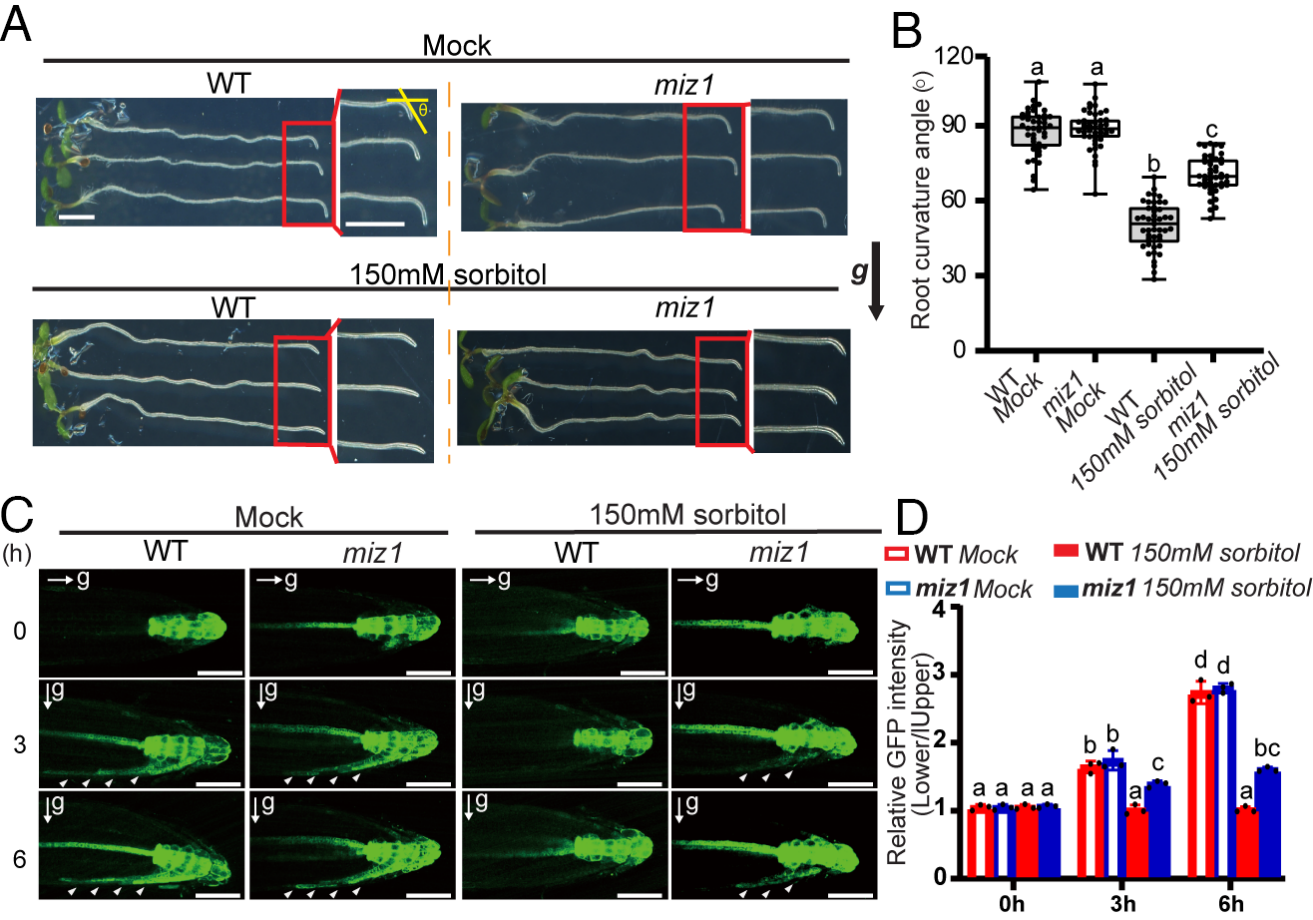
MIZ1-dependent effect of osmotic stress on gravity-induced asymmetric auxin distribution and root gravitropic bending. (*A*) Phenotypic analysis of root gravitropism of 7-d-old *Arabidopsis* WT and *miz1* mutant with no treatment (Mock) or 150 mM sorbitol treatment. Roots were gravistimulated for 6 h following a 90° reorientation. (Scale bars, 2 mm.) (*B*) Quantification of the root bending in (*A*) for each group (Three biological replicates for each group, n ≥ 10 roots for each replicate). Each circle represents the measurement of an individual root. Boxplots span the first to the third quartiles of the data. Whiskers indicate the minimum and maximum values. A line in the box represents the mean. (*C*) Auxin redistribution in roots of WT and *miz1* mutant with no treatment (Mock) or 150 mM sorbitol treatment upon gravistimulation, as indicated by the auxin-responsive reporter *DR5rev::GFP*. Images were taken separately after 0, 3, and 6 h of gravistimulation. The white arrows indicate the *DR5rev::GFP* signal at the lateral root flanks after gravistimulation. (Scale bars, 50 μm.) (*D*) Statistical analysis of the *DR5rev::GFP* intensity ratio between the lower and upper lateral root flanks in (*C*). Error bars represent SE from three biological replicates. No fewer than five roots for each replicate. Significant differences were determined by one-way ANOVA followed by Tukey’s multiple comparisons test, with a significance level of *P* < 0.001.

Next, we explored whether MIZ1 attenuates root gravitropism under drought stress. The D-sorbitol applied in the split-agar/sorbitol system is a widely used approach to mimic drought stress (20, 28, 29). In agreement with a previous report (20), following gravistimulation, both the *miz1* mutant and wild-type (WT) exhibited comparable root gravitropism under standard conditions (Fig. 2*A* and *SI Appendix*, Fig. S1). However, under low environmental Wp (osmotic stress) generated by 150 mM sorbitol treatment, root gravitropism in the WT was inhibited (Fig. 2 *A* and *B*), indicating that *Arabidopsis* root gravitropism is negatively regulated by osmotic stress. Notably, following 150 mM sorbitol treatment, the *miz1* mutant displayed much faster root gravitropism relative to the WT (Fig. 2*A*), even though the root growth rates between the WT and *miz1* mutant were comparable (*SI Appendix*, Fig. S2). These findings demonstrate that *Arabidopsis* roots rapidly recognize a decrease in environmental Wp and subsequently attenuate root gravitropic growth, thereby promoting root hydrotropism. Furthermore, this adaptive regulation depends on MIZ1 activity.

The central mechanism underlying root gravitropism is the asymmetrical auxin redistribution along the root. To determine whether the osmotic stress-induced inhibition of root gravitropism is related to auxin redistribution, we tested the auxin-responsive reporters *DR5rev::(GFP)* green fluorescent protein (30) and *DR5rev::3xVENUS-N7* (31). Sorbitol treatment significantly inhibited gravity-induced asymmetric DR5 expression (Fig. 2 *C* and *D* and *SI Appendix*, Fig. S3), explaining the reduced root gravitropism (Fig. 2*A*). Additionally, another auxin sensor, *R2D2* (32), confirmed the defect in gravity-induced asymmetric auxin response after sorbitol treatment (*SI Appendix*, Fig. S4). Next, we introduced *DR5rev::GFP*, *DR5rev::3xVENUS-N7*, and *R2D2* markers into the *miz1* mutant. In contrast to the WT roots, the *miz1* mutant showed strong asymmetric expression of auxin-responsive genes following gravistimulation, even after sorbitol treatment (Fig. 2*C* and *SI Appendix*, Figs. S3 and S4). These results suggest that MIZ1 is required for the inhibitory effect of drought stress on asymmetric auxin distribution during root gravitropism.

Given that the asymmetric ABA response has been implicated in root hydrotropism and that ABA negatively regulates root gravitropism (33, 34), we tested whether ABA signaling is involved in the MIZ-mediated inhibition of asymmetric auxin transport and root gravitropism under osmotic stress. The application of 1 μM ABA markedly inhibited the asymmetric auxin response and root gravitropism in both WT and *miz1* mutant in a comparable way (*SI Appendix*, Fig. S5 *A*–*D*). Additionally, in the growth medium containing 150 mM sorbitol, whether treated with 1 μM ABA or not, the *miz1* mutant stably exhibited significantly faster root gravitropism and asymmetric auxin transport than the WT (Fig. 2 and *SI Appendix*, Fig. S5).

Taken together, our observations show that drought stress, as mimicked by sorbitol treatment, attenuates asymmetric auxin distribution and the resulting bending during root gravitropism and that MIZ1 mediates this effect, thus enhancing root hydrotropism. It appears that this MIZ1-mediated inhibition of root gravitropism under drought stress does not involve ABA signaling.

### Sorbitol Treatment Targets PINs’ Function in Auxin Transport to Promote Root Hydrotropism via MIZ1.

Asymmetric auxin flow is a crucial mechanism during root gravitropism that occurs via a two-step process: Following gravistimulation, sensed by the sedimentation of statoliths (amyloplasts), PIN3 is polarized to the lower side of root columella cells to divert auxin flow downward. This step initiates the asymmetric auxin distribution in the root tip (11). Subsequently, PIN2, which is polarly localized at the shootward side of root epidermal cells (3), amplifies the initial asymmetric auxin flow and ultimately establishes the asymmetric auxin distribution from the root tip to the elongation zone.

To explore, in which step MIZ1 takes part to regulate drought stress-inhibited auxin asymmetric redistribution, we first analyzed amyloplasts in roots using modified pseudo-Schiff propidium iodide (mPS-PI) staining. The results showed that both *miz1* and WT contain similar amounts of amyloplasts in the root apex (*SI Appendix*, Fig. S6*A*). Similarly, after sorbitol treatment, both WT and *miz1* displayed comparably decreased amounts of amyloplasts in the root apex (*SI Appendix*, Fig. S6 *B* and *C*). These results suggest that MIZ1 does not regulate the root-apex localized amyloplast accumulation to mediate the osmotic stress-induced inhibition of auxin asymmetric distribution.

**Fig. 3.**
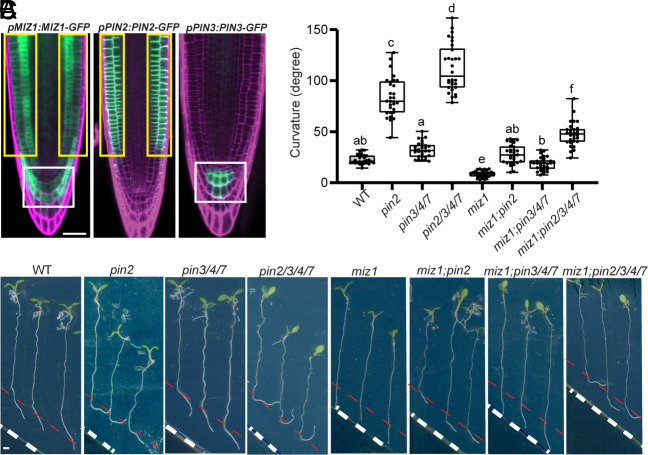
MIZ1 effect on osmotic stress-induced PIN inactivation for promotion of root hydrotropism. (*A*) Expression patterns of *MIZ1*, *PIN2*, and *PIN3* in *Arabidopsis* roots. (Scale bars, 50 μm.) (*B*) Hydrotropic responses of representative WT, *pin2*, triple mutant *pin3/4/7*, quadruple mutant *pin2/3/4/7,* and *pin* mutants in the *miz1* mutant background, including *miz1*, *miz1;pin2*, *miz1;pin3/4/7*, and *miz1;pin2/3/4/7*, using the split agar/sorbitol system. (Scale bars, 1 mm.) (*C*) Measurement of root hydrotropic growth curvatures for plants in (*B*). Three biological replicates for each line, n ≥ 10 roots for each replicate. Each circle represents the measurement of an individual root. Boxplots span the first to the third quartiles of the data. Whiskers indicate minimum and maximum values. A line in the box represents the mean. Significant differences were determined by one-way ANOVA followed by Tukey’s multiple comparisons test, with a significance level of *P* < 0.001.

Next, we investigated whether MIZ1’s action on asymmetric auxin flow during root gravitropism involves PIN auxin transporters. Expression pattern analysis of MIZ1 and PIN proteins revealed, in agreement with previous reports (35), that MIZ1 is expressed in the root epidermis, lateral root cap, and columella cells, coinciding with the expression pattern of PIN2 and PIN3 in *Arabidopsis* roots (Fig. 3*A*). This supports a potential interplay between MIZ1 and PIN2/PIN3 in root hydrotropism.

Therefore, we examined the root hydrotropic growth of three *Arabidopsis* loss-of-function *pin* mutants, including *pin2*, triple mutant *pin3/4//7*, and quadruple mutant *pin2/3/4/7*. In *Arabidopsis*, the *PIN3* clade contains three genes (*PIN3*, *PIN4*, and *PIN7*), which function redundantly in mediating auxin flow in columella cells. Hence, the triple mutant *pin3/4/7* instead of their single mutants was used for hydrotropic growth analysis. Compared to WT, both *Arabidopsis pin2* and *pin3/4/7* showed significantly enhanced root hydrotropism, as evidenced by their prominently increased hydrotropic bending curvature in the split-agar/sorbitol system (Fig. 3*B*). The *pin2/3/4/7* displayed an even greater hydrotropic bending angle than either *pin2* or *pin3/4/7* alone (Fig. 3 *B* and *C*). Together, these findings imply that PIN2 and PIN3/PIN4/PIN7 activity is involved in root hydrotropism.

Afterward, to test whether PIN functions are linked to the MIZ1’s action on root hydrotropism, we crossed *miz1* with these *pin* mutants. Remarkably, both *miz1;pin2* and *miz1;pin3/4/7* mutants exhibited significantly increased root hydrotropic growth compared to *miz1* mutant alone (Fig. 3 *B* and *C*), suggesting that nullification of *PIN2* or *PIN3/PIN4/PIN7* function in the *miz1* mutant can rescue its defective root hydrotropism. Moreover, *miz1;pin2/3/4/7* displayed much stronger root hydrotropism than *miz1;pin2* and *miz1;pin3/4/7* mutants (Fig. 3*B*).

Collectively, this genetic analysis suggests that MIZ1’s action on root hydrotropism involves PIN-mediated auxin transport.

### Sorbitol Treatment Erodes PIN Polarity via MIZ1.

**Fig. 4.**
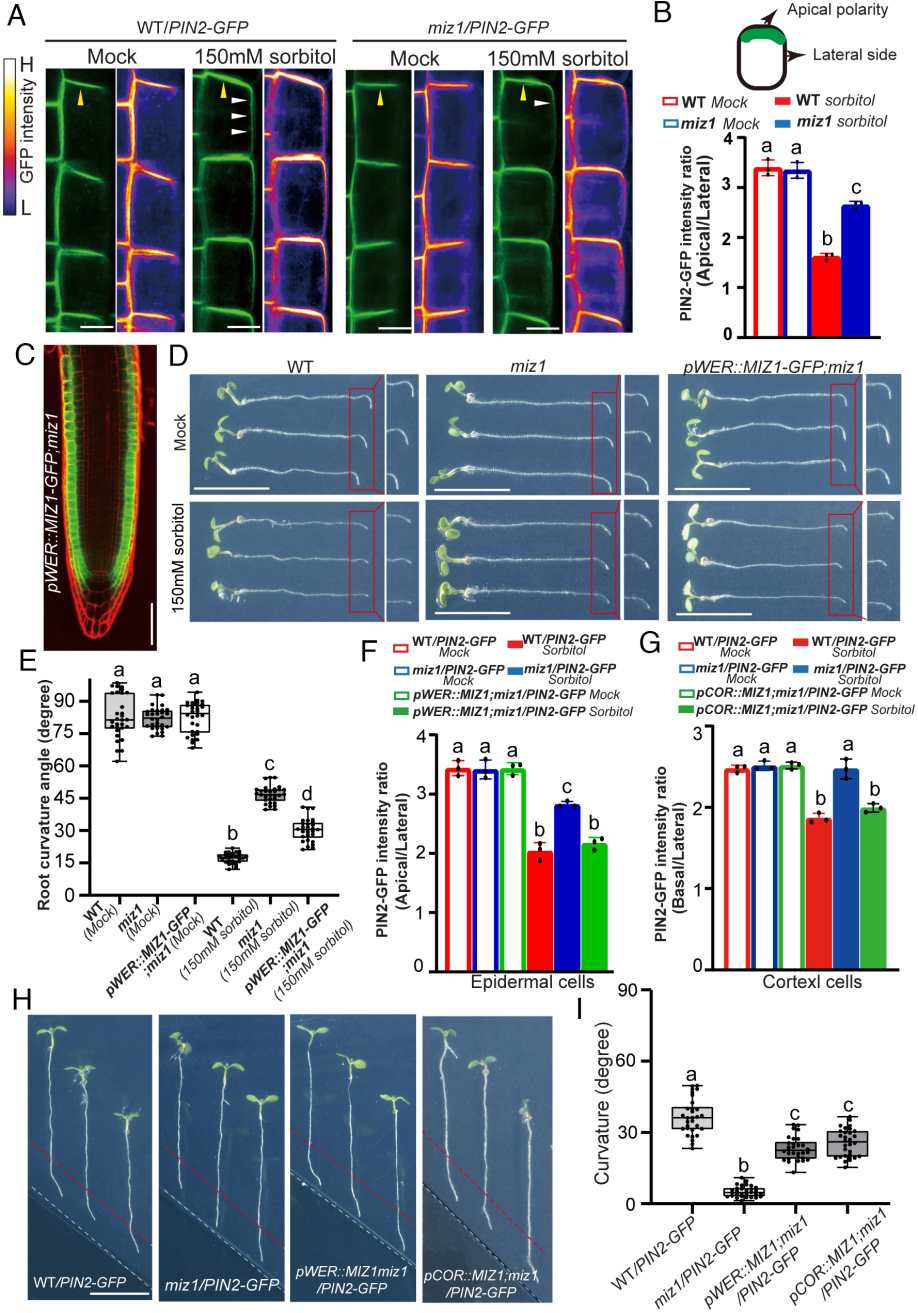
MIZ1-dependent effect of osmotic stress on PIN polarity to inhibit root gravitropism and promote root hydrotropism. (*A*) The PIN2 polarity at the PM of root epidermal cells (specifically atrichoblasts) in WT and *miz1* mutant plants, without (Mock) or with 150 mM sorbitol treatment. Yellow arrowheads indicate the apical signal of PIN2-GFP in root epidermal cells, while white arrows indicate the PIN2-GFP signal at the lateral side of epidermal cells, demonstrating the decreased PIN2 polarity in WT root epidermal cells treated with 150 mM sorbitol. (Scale bars, 10 μm.) The spectrum lookup table (LUT) is used to represenvt the GFP signal, with the intensity scale shown at the *Right* of the images (H, high; L, Low). (*B*) Quantitative analysis of the PIN2-GFP signal intensity between apical and lateral sides of atrichoblasts in (*A*). Error bars represent SE from three biological replicates. No fewer than 20 atrichoblast cells from five roots were analyzed for each replicate. (*C*) Specific expression of *MIZ1-GFP* in root epidermal cells of *pWER::MIZ1-GFP;miz1 Arabidopsis* transgenic line. (Scale bars, 50 μm.) (*D*) Phenotypic analysis of root gravitropism in 7-d-old *Arabidopsis* WT, *miz1* mutant, and *pWER::MIZ1-GFP/miz1* line, with or without 150 mM sorbitol treatment. Roots were gravistimulated for 6 h following a 90° reorientation. (Scale bars, 1 cm.) (*E*) Quantification of root bending in (*D*) for each group (three biological replicates per group, n ≥ 10 roots for each replicate). Each circle represents the measurement of an individual root. Boxplots span the first to the third quartiles of the data. Whiskers indicate minimum and maximum values. The line within the box represents the median. (*F*) Quantitative analysis of the PIN2-GFP ratio between apical and lateral sides of atrichoblasts in the *pWER::MIZ1;miz1/PIN2-GFP* line shown in (*SI Appendix*, Fig. S7*A*). Error bars represent SE from three biological replicates. No fewer than 20 atrichoblast cells from five roots were analyzed for each replicate. (*G*) Quantitative analysis of the PIN2-GFP ratio between basal and lateral sides of cortex cells in the *pCOR::MIZ;miz1/PIN2-GFP* line shown in (*SI Appendix*, Fig. S7*D*). (*H*) Hydrotropic responses of WT*/PIN2-GFP*, *miz1/PIN2-GFP*, *pWER::MIZ1;miz1/PIN2-GFP*, and *pCOR::MIZ1;miz1/PIN2-GFP* lines in the split agar/sorbitol system. (Scale bars, 1 cm.) (*I*) Measurement of root hydrotropic growth curvatures of plants in (*H*) for each line (three biological replicates for each line, n ≥ 10 roots for each replicate). Each circle represents the measurement of an individual root. Boxplots span the first to the third quartiles of the data. Whiskers indicate minimum and maximum values. A line in the box represents the mean. Significant differences were determined by one-way ANOVA followed by Tukey’s multiple comparisons test, with a significance level of *P* < 0.001.

PIN polarity at the plasma membrane (PM) is the key factor determining the directionality of auxin flow (10). Hence, we investigated the effect of osmotic stress on PIN polarity. Under normal conditions (without sorbitol treatment), the PIN2 polarity in root epidermal cells of both the WT and *miz1* mutant showed no significant difference, with the PIN2-GFP signal mainly concentrated at the apical side of the epidermal cells (Fig. 4 *A* and *B*). However, after the sorbitol treatment, the PIN2 polarity was less pronounced, as evidenced by the enhanced GFP signal at the lateral side of root epidermal cells (Fig. 4*A*). In contrast, the *miz1* mutant maintained a pronounced apical PIN2-GFP signal, with less spread to the lateral cell sides following sorbitol treatment (Fig. 4 *A* and *B*).

To confirm that MIZ1 regulates PIN2 polarity in root epidermal cells under osmotic stress, we used the *WER* promoter to drive the expression of *MIZ1* or *MIZ1-GFP* specifically in root epidermal cells of the *miz1* mutant. The *pWER::MIZ1-GFP;miz1* line revealed that when *MIZ1-GFP* was expressed specifically in root epidermal cells (Fig. 4*C*), it partially rescued the gravitropic phenotype of the *miz1* after sorbitol treatment, as manifested by a substantially decreased root gravitropism compared to the *miz1* mutant (Fig. 4 *D* and *E*). Consistently, the PIN2-GFP in *pWER::MIZ1;miz1* line after sorbitol treatment showed a polarity pattern similar to the WT, rather than the *miz1* mutant (Fig. 4*A*), meaning the decreased apical polarity in epidermal cells was rescued (Fig. 4*F* and *SI Appendix*, Fig. S7*A*). Additionally, the expression of *MIZ1* and *MIZ1-GFP* in root epidermal cells, driven by the *WER* promoter, rescued the hydrotropic growth defects in the *miz1* mutant (Fig. 4 *H* and *I*). These results highlight the role of MIZ1 in root epidermis cells in inhibiting PIN2 polarity and gravitropism in response to osmotic stress, thereby antagonistically balancing the root gravitropism and root hydrotropism.

In addition to apical localization in root epidermal cells, PIN2 is also expressed in cortex cells, where it is localized at the basal (rootward) side to regulate root gravitropism (36). Our results showed that 150 mM sorbitol treatment decreased the basal polarity of PIN2 in the cortex cells of WT *Arabidopsis*, with a strong GFP signal detected at the lateral sides (*SI Appendix*, Fig. S7 *B* and *C*). In contrast, this regulatory effect was not observed in the *miz1* mutant (*SI Appendix*, Fig. S7*C*). To further confirm the role of MIZ1 in regulating PIN2 polarity dynamics in cortex cells in response to osmotic stress, we utilized the *COR* promoter to specifically express the *MIZ1* gene in the root cortex cells of the *miz1* mutant carrying the *PIN2-GFP* construct. Consistent with previous reports (28), the cortex cell-specific expression of *MIZ1* in *Arabidopsis* roots rescued the defective root hydrotropism of the *miz1* mutant (Fig. 4 *H* and *I*). Furthermore, in contrast to the *miz1* mutant, the PIN2-GFP in the *pCOR::MIZ1;miz1* line exhibited a significant reduction in its basal (rootward) polarity in the cortex cells under sorbitol treatment (Fig. 4*G* and *SI Appendix*, Fig. S7*D*), closely mirroring the PIN2-GFP polarity in WT plants subjected to the same treatment (*SI Appendix*, Fig. S7*B*). These findings strongly suggest that MIZ1 also dynamically regulates the function of PIN2 in root cortex cells in response to osmotic stress.

Likewise, PIN3 in both WT and *miz1* mutant was repolarized preferentially to the lower sides of the columella cells following gravistimulation under normal conditions (*SI Appendix*, Fig. S8 *A*–*C*). However, when treated with sorbitol, gravity-induced PIN3 repolarization in the WT was inhibited, showing no distinguishable polarity (*SI Appendix*, Fig. S8). In contrast, PIN3 in the *miz1* was still repolarized and accumulated visibly at the lower sides of columella cells (*SI Appendix*, Fig. S8).

Taken together, our results indicate that MIZ1 reduces PIN2 polarity and inhibits gravity-induced PIN3 repolarization under drought stress, thus interfering with asymmetric auxin redistribution for root gravitropism, ultimately promoting root hydrotropism.

### Sorbitol Treatment Affects PIN2 Subcellular Trafficking via MIZ1.

As subcellular trafficking is the primary mechanism in the establishment and maintenance of PIN2 polarity (37), we next tested the effect of osmotic stress on PIN2 trafficking. The vesicle-trafficking inhibitor Brefeldin A (BFA) inhibits protein recycling and causes the accumulation of PM-localized proteins into intracellular aggregates known as BFA bodies. This effect is reversible; after BFA washout, the BFA bodies disappear as proteins return to the PM (38). Thus, BFA is a convenient tool for studying PIN2 trafficking.

**Fig. 5.**
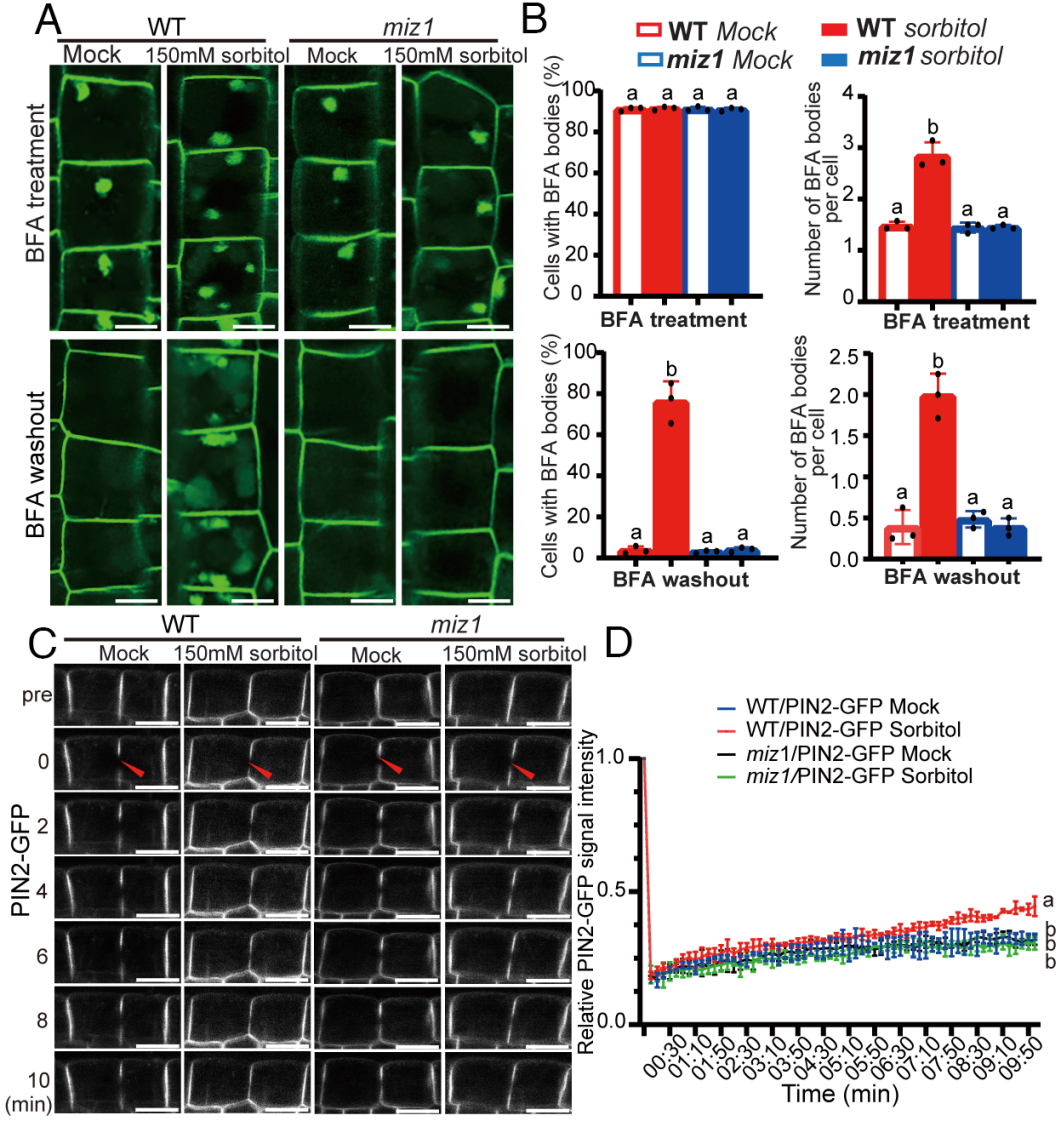
MIZ1-dependent effect of osmotic stress on PIN2 subcellular trafficking and lateral diffusion to inhibit root gravitropism and promote root hydrotropism. (*A*) Visualization of PIN2-GFP-containing BFA bodies in *Arabidopsis* root of WT and *miz1* mutant, treated with 50 μM BFA for 1 h, either following a 1-h treatment with 150 mM sorbitol or no treatment (Mock) (*Upper*). Additionally, PIN2-GFP-labeled BFA bodies were observed in WT and *miz1* mutant after a 2-h BFA washout (*Lower*). (Scale bars, 10 μm.) (*B*) Quantification of cells with BFA bodies and the number of BFA bodies per cell observed in (*A*). (*C*) FRAP dynamics of PIN2-GFP in root epidermal cells (specifically atrichoblasts) of WT (*Col-0*) and *miz1* mutant, with or without 150 mM sorbitol treatment. (Scale bars, 10 μm.) (*D*) Quantitative analysis of the fluorescence recovery rate of PIN2-GFP after photobleaching in (*C*). Error bars represent SE from three biological replicates. No fewer than 20 cells from five roots were analyzed for each replicate. Significant differences were determined by one-way ANOVA (*B*) or two-way ANOVA (*D*) followed by Tukey’s multiple comparisons test, with a significance level of *P* < 0.001.

Our results showed that treatment with 50 μM BFA for 1 h did not result in a significant difference in the number of PIN2-containing BFA bodies or the percentage of cells with BFA bodies between WT and *miz1* mutant (Fig. 5*A*). However, exposure of WT roots to 150 mM sorbitol led to a substantial increase in the accumulation of PIN2-containing BFA bodies compared to untreated controls (Fig. 5*A*). Importantly, this osmotic stress-induced effect on PIN2 trafficking was not observed in the *miz1* mutant, as evidenced by the significantly lower accumulation of PIN2-containing BFA bodies in *miz1* mutant root cells compared to WT when treated with sorbitol (Fig. 5 *A* and *B*). Consistent with these observations, after a 2-h BFA washout, PIN2-BFA bodies disappeared in sorbitol-treated *miz1* mutant roots, similar to both WT and *miz1* mutant cells without sorbitol treatment (Fig. 5*A*). In contrast, PIN2-BFA bodies persisted in the root cells of WT after sorbitol treatment (Fig. 5 *A* and *B*). These findings suggest that osmotic stress modulates BFA-sensitive trafficking of PIN2 via MIZ1, which in turn explains the observed decrease in PIN2 polarity, asymmetric auxin accumulation, and gravitropic bending (Figs. 2 *C* and 4*A*).

### Sorbitol Treatment Enhances PIN Lateral Diffusion via MIZ1.

The lateral diffusion rate of polar proteins within the PM plays a crucial role in maintaining and establishing their polarity in the PM (37, 39). To evaluate the PIN lateral diffusion rate in epidermal and cortex cells of WT and *miz1* mutant, we carried out the fluorescence recovery after photobleaching (FRAP) assays. Our results showed that the fluorescence recovery rate of PIN2-GFP in WT was comparable to that in the *miz1* mutant under standard conditions (Fig. 5*C* and *SI Appendix*, Fig. S9). However, when treated with sorbitol, the recovery rate of PIN2-GFP in epidermal and cortex cells of WT was dramatically increased (Fig. 5*C* and *SI Appendix*, Fig. S9), likely contributing to the decreased PIN2 polarity observed (Fig. 4*A*). In contrast, the FRAP of PIN2-GFP in epidermal and cortex cells of the *miz1* mutant was much slower than that in the WT following sorbitol treatment (Fig. 5 *C* and *D* and *SI Appendix*, Fig. S9), suggesting that MIZ1 is required for the osmotic stress-induced promotion of PIN2 lateral diffusion within the PM, which in turn reduces PIN2 polarity.

Subsequently, we examined the diffusion rate of PIN3 in the PM of columella cells. Similarly, FRAP of PIN3 in WT was comparable to that in the *miz1* mutant under standard conditions (*SI Appendix*, Fig. S10). However, after sorbitol treatment, the FRAP of PIN3 in the WT was markedly increased compared to that in the *miz1* mutant (*SI Appendix*, Fig. S10). These results suggest that MIZ1 enhances the lateral diffusion of PIN3 within the PM of root columella cells in response to osmotic stress.

Furthermore, to ascertain whether MIZ1-mediated regulation of protein dynamics under osmotic stress is specific to PIN proteins or represents a general mechanism affecting other PM-localized proteins, we introduced marker lines carrying other potentially polarly localized proteins, including *pAUX1::AUX1-YFP*, *pPEN3::PEN3-GFP,* and *35S::BOR4-GFP*, into the *miz1* mutant. FRAP experiments demonstrated that the recovery rates of the PM-localized proteins AUX1, PEN3, and BOR4 were comparable in both WT and *miz1* mutant backgrounds, irrespective of treatment with 150 mM sorbitol (*SI Appendix*, Fig. S11). These findings suggest that the MIZ1-mediated lateral diffusion for PIN proteins within the PM in response to osmotic stress is relatively specific to PIN proteins.

Collectively, our findings show that MIZ1 mediates the drought stress-induced lateral diffusion of PIN auxin transporters in the PM, likely interfering with the maintenance of PIN2 polarity in epidermal/cortex cells and PIN3 polarization in columella cells. The impaired PIN polarity leads to defective gravity-induced auxin redistribution, thereby inhibiting root gravitropism and promoting root hydrotropism.

### The MIZ1-PIN Module Is Involved in Hydrotropic Root Growth for Drought Avoidance in Soil.

**Fig. 6.**
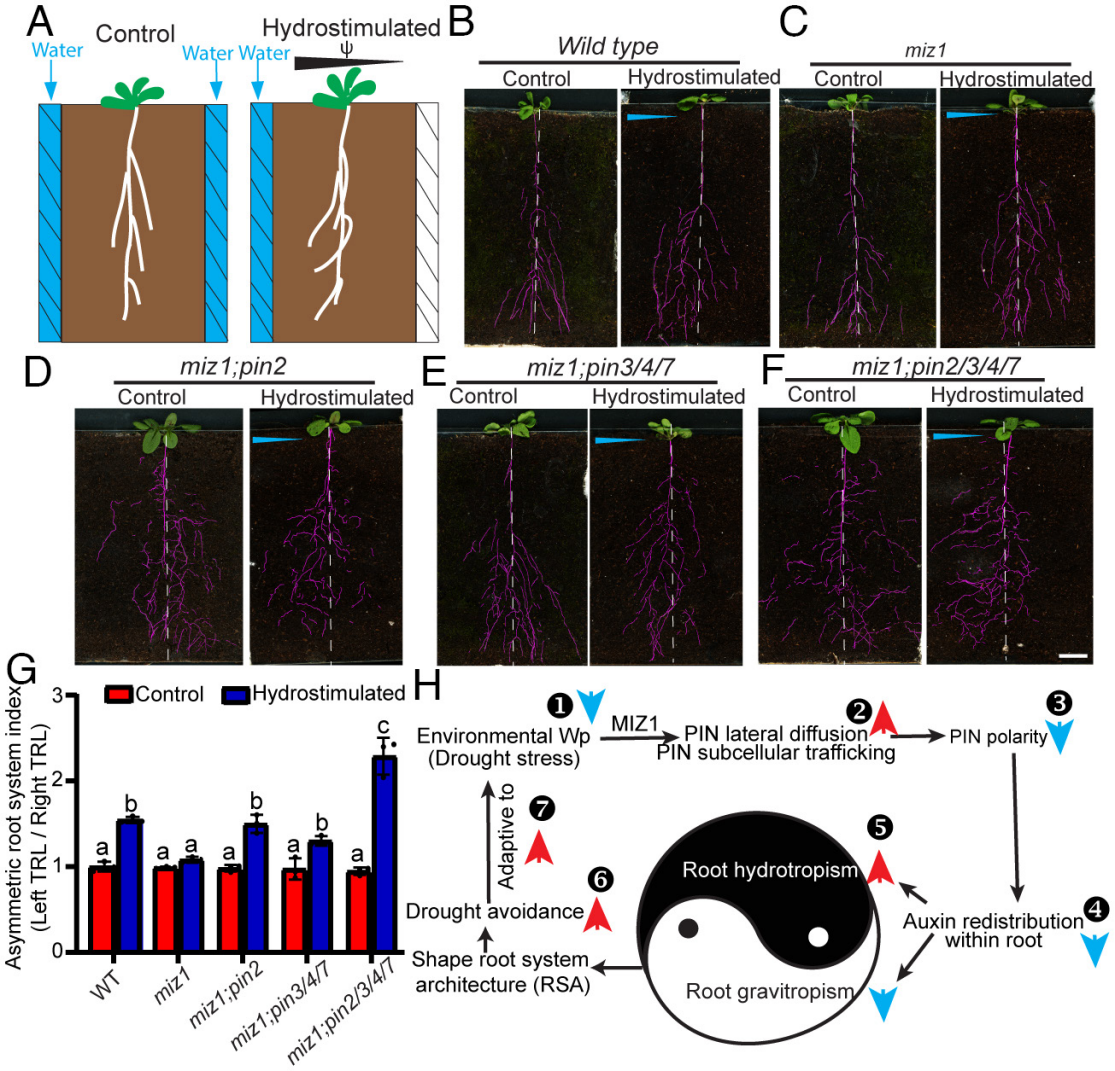
The MIZ1-PIN regulatory module is involved in hydrotropic root growth for drought avoidance. (*A*) Diagram of the experimental setup used to study the *Arabidopsis* RSA under hydrostimulation. (*B*–*F*) Representative plant roots used for experimental analysis in the absence (Control) and presence of hydrostimulation. (*B*) WT, (*C*) *miz1*, (*D*) *miz1;pin2*, (*E*) *miz1;pin3/4/7*, and (*F*) *miz1;pin2/3/4/7* mutant. (Scale bars, 2 cm.) (*G*) Quantitative analysis of the asymmetric index of the root system in (*B*–*F*) by calculating the ratio between left total root length (TRL) and right TRL. Error bars represent SE from three biological replicates, each with three plants. Significant differences were determined by one-way ANOVA followed by Tukey’s multiple comparisons test, with a significance level of *P* < 0.001. (*H*) Schematic of the proposed model. External Wp is translated into endogenous auxin signal in a MIZ1-dependent manner to modulate the trade-off between gravitropism andhydrotropism. Specifically, a decrease in external Wp (osmotic stress) promotes both the subcellular trafficking and lateral diffusion rate of PIN2, leading to a reduction in PIN2 polarity. This, in turn, diminishes auxin redistribution efficiency along the root longitude axis, thereby attenuating root gravitropism while enhancing root hydrotropism. Consequently, increased root hydrotropism shapes the RSA to favor growth in moister soil, thereby promoting drought avoidance. This adaptive strategy endows the roots with high growth plasticity to evade drought, enhancing the plant’s overall adaptability to drought stress. The red and blue arrows represent the promoting and reducing effects, respectively.

Root tropisms determine root system architecture (RSA), contributing to environmentally adaptive growth and ultimately plant performance (40–43). To complement our somehow artificial split/agar-sorbitol system, we established experiments with moisture gradients in the soil to test the role of MIZ1 and PINs in shaping the RSA for drought avoidance (Fig. 6*A*).

In the control chamber with water applied to both sides, the WT exhibited an almost symmetrical RSA between the two sides of the chamber. However, in the chamber with water applied only to one side, the WT RSA showed a strong asymmetry between the moist and dry sides of the chamber (Fig. 6*B*). In contrast to the WT, the RSA of the *miz1* mutant did not show such an asymmetric RSA in the hydrostimulated chamber (Fig. 6*C*), consistent with a previous report (9).

To investigate the role of PINs in the establishment of asymmetric RSA in response to water gradient, we analyzed the *pin2*, *pin3/4/7*, and *pin2/3/4/7* mutants. Similar to WT, the RSA of these *pin* mutants was symmetric in the soil without hydrostimulation (*SI Appendix*, Fig. S12 *A*–*D*). However, in the hydrostimulated chamber, the *pin* mutants, in particular the *pin2/3/4/7* quadruple mutant, displayed dramatically higher root density on the moist side compared to WT (*SI Appendix*, Fig. S12 *A*–*E*). These results suggest that inactivation of the four PINs in *Arabidopsis* not only enhances root hydrotropism (Fig. 3*B*) but also substantially promotes the hydrotropic growth of secondary roots, resulting in a strong asymmetric RSA toward the moist soil. Moreover, following hydrostimulation, in contrast to the *miz1* mutant (Fig. 6*C*), the *miz1;pin2, miz1;pin3pin4pin7,* and *miz1;pin2pin3pin4pin7* mutants displayed remarkably more asymmetric RSA, with a very high root density at the moist side of the hydrostimulated chamber (Fig. 6 *D*–*G*). These results suggest that the MIZ1-PIN regulatory module controls root hydrotropic growth to determine RSA for drought avoidance.

## Discussion

In this work, we have uncovered a key regulatory mechanism that governs adaptive tropic growth and RSA under conditions of simultaneous gravity stimulation and asymmetric water availability. Our observations demonstrate that gravitropism and hydrotropism act antagonistically and that drought stress attenuates gravitropism, thereby removing its antagonizing effect and enabling more efficient root growth toward moisture. We also show that MIZ1, a master regulator of hydrotropism, mediates this drought stress-induced attenuation of gravitropism, providing insights into a cellular mechanism underlying this action.

Tropic responses can be broadly divided into three biological processes: i) Perception of environmental cues; ii) transduction of the perceived signal; and iii) growth response (5, 44). Here, we refine the model of root hydrotropism and assign a new role to the central root hydrotropism regulator, MIZ1. We found that a decrease in environmental Wp (emulated by the addition of sorbitol to the medium) enhances the lateral diffusion rate of PIN auxin transporters and modulates their trafficking, leading to defects in PIN polarity at the PM. The reduction in PIN polarity attenuates directional auxin transport, thus inhibiting auxin redistribution within the root and root gravitropic growth, while promoting hydrotropism. All these drought stress-induced effects strictly depend on MIZ1. Similarly, the establishment of asymmetric RSA in response to a water gradient in the soil depends on the activities of PIN and MIZ1 (Fig. 6*H*). These findings suggest that root tropic responses to water and gravity are closely linked through the MIZ1-PIN regulatory module. Additionally, it has been well-established that auxin and cytokinin antagonistically regulate root gravitropic growth to control vertical growth of the lateral roots (41, 45), ultimately determining the formation of shallow versus deep root architecture for plant drought and waterlogging tolerance (42, 43). Our findings, combined with the previous report that cytokinin can also be redistributed between root sides following hydrostimulation (20), suggest that the auxin and cytokinin also antagonistically regulated root hydrotropism to define the lateral asymmetry of RSA (*SI Appendix*, Fig. S13). This mechanism endows plant roots with high plasticity for efficient water tracking and drought avoidance.

When plants are cultivated outside Earth’s gravitational field, their roots experience a loss of gravitropism. By comprehending the mechanistic connection between gravitropism and other plant tropisms, such as root hydrotropism, we can predict the root response to water and other environmental stimuli in the absence of gravity. This knowledge will also contribute to improving plant adaptability to zero- or microgravity environments beyond Earth.

The unresolved issues in our model pertain to the mechanism underlying the perception and translation of Wp into directional growth, as well as the molecular role of the MIZ1 protein and its impact on the lateral diffusion and trafficking of PIN transporters. Even though the precise operational model of hydrotropism remains enigmatic, we provide a key piece of this puzzle: MIZ1 regulates the PIN-mediated auxin redistribution in roots in response to changes in environmental water availability. This, in turn, modifies plant gravitropism and hydrotropism, enabling roots to seek out water and avoid drought.

## Materials and Methods

### Plant Materials and Growth Conditions.

The *Arabidopsis* loss-of-function *pin* mutants (*pin2, pin3/4/7,* and *pin2/3/4/7*) and *Arabidopsis* transgenic lines (*DR5rev::GFP*, *DR5rev::3XVENUS-N7, R2D2, pPIN2::pPIN2-GFP*, *pPIN3::PIN3-GFP*, p*MIZ1::MIZ1-GFP*, *35S::BOR4-GFP*, *pPEN3::PEN3-GFP*, *pAUX1::AUX1-YFP*) used in this study were previously described (36, 37, 46–48); the loss-of-function mutant *miz1* (SALK_076560) was also previously reported (8). The mutants *miz1;pin2*, *miz1;pin3/4/7*, and *miz1;pin2/3/4/7* were constructed by crossing *pin2, pin3/4/7,* and *pin2/3/4/7* as the male parent with *miz1* as the female parent. Similarly, the *miz1;DR5rev::GFP*, *miz1;DR5rev::3XVENUS-N7, miz1;R2D2, PIN2::PIN2-GFP*, *miz1;PIN3::PIN3-GFP*, *miz1;35S::BOR4-GFP*, *miz1;PEN3::PEN3-GFP,* and *miz1;AUX1::AUX1-YFP* were generated by crossing these transgenic lines carrying the fluorescent reporter with the *miz1* mutant. Seeds were surface-sterilized by chlorine vapor, sown on 1/2 Murashige-Skoog (½ MS) medium supplemented with 1% sucrose and 1% agar and grown in vitro under long-day conditions. For the gravistimulation assay, 7-d-old seedlings were transferred to a standard medium, 150 mM D-sorbitol-containing medium, 1 μM ABA-containing medium, or 150 mM D-sorbitol plus 1 μM ABA-containing medium, following 1 h of acclimation at the original growth orientation before the plates were rotated 90°.

### Simulating Plant Growth in Microgravity/Weightlessness Using a Clinostat.

Roots were hydrotropically stimulated using the diagonal split-agar/sorbitol system (22). Briefly, plants were placed on 1% solidified agar, right-angle triangle cut ½ MS medium, such that the root tips were 5 mm from the hypotenuse edge, followed by attachment of an 800 mM D-sorbitol containing medium triangle to form a square, allowing the sorbitol to diffuse into the upper triangle. To determine the root hydrotropic bending, we photographed the square plates using a digital scanner (EPSON Perfection V370) and analyzed the images using ImageJ software (Wayne Rasband, NIH, USA).

To simulate root hydrotropism under the weightless or microgravity environment, ten 7-d-old seedlings were transferred into the split-agar/sorbitol system. Then, the square plates were sealed with aluminum foil to ensure that the seedlings were grown under dark conditions, followed by the attachment of this system to a slowly rotating clinostat (1 rpm) developed in our lab. In total, around 30 seedlings of each sample were analyzed in each experimental procedure. Pictures were taken after 24 h of exposure to each condition. Root hydrotropic bending curvatures after 24 h of clinorotation were measured with ImageJ software (https://imagej.net/Fiji).

### Starch Staining.

The starch granules and cell walls in *Arabidopsis* root tips (7-d-old) were stained using the mPS-PI method and imaged with a confocal microscope as previously described (49). In brief, whole seedlings were fixed in 50% methanol/10% acetic acid at 4 °C for up to 24 h. The tissue was rinsed briefly with ddH_2_O and incubated in 1% periodic acid at room temperature for 40 min. The tissue was then rinsed twice with ddH_2_O and incubated in Schiff reagent with propidium iodide (100 mM sodium metabisulfite, 0.15 N HCl, and 100 mg/mL PI) for 2 h until the plants were visibly stained. More than three samples were transferred onto microscope slides and covered with chloral hydrate solution (4 g chloral hydrate, 1 mL glycerol, and 2 mL water). The slides were kept overnight at room temperature, after which excess chloral hydrate was removed. The seedlings were mounted in Hoyer’s solution (30 g gum arabic, 200 g chloral hydrate, 20 g glycerol, and 50 mL water). The slides were left undisturbed for at least 3 d before observation (excitation 488 nm, emission 520 to 720 nm).

### Vector Construction and Plant Transformation.

For epidermal cell type–specific genetic complementation analysis of the *miz1* phenotype, a 4.3-kb promoter of *WER* (*WEREWOLF*), a 2-kb promoter of *COR*, and the coding sequence (CDS) of *MIZ1* or *MIZ1-GFP* were amplified from WT and the *pMIZ1*::*MIZ1-GFP* transgenic line, respectively. The promoter and CDS sequences were then separately cloned into the Gateway entry vector pDONRP4P1r and pDONR221 via BP reaction, and subsequently, they were fused and cloned into the Gateway destination vector pB7m24GW.3 using Gateway LR reaction. The resulting constructs were transformed into the *miz1* mutant. Transgenic *Arabidopsis* plants were generated by the floral dip method and subsequently selected on solid half-strength MS medium plates containing 50 mg/L of the appropriate antibiotics.

### BFA Treatment and Washout.

For the BFA treatment experiments, 6-d-old *Arabidopsis* seedlings expressing PIN2-GFP fluorophore-tagged proteins were incubated in liquid 1/2 MS medium containing at a final concentration of 50 μM BFA for 1 h. This incubation was performed immediately following a 1-h treatment with 150 mM sorbitol or no treatment for control samples. For the BFA washout experiments, the seedlings were rinsed and then maintained in liquid 1/2 MS medium for 2 h. Cells (n = 20 to 30) from 15 plants of three replications were used for BFA content measurement and statistical analysis, and representative pictures are presented to illustrate the results.

### Confocal Imaging and Image Analysis.

For confocal imaging of plants carrying *DR5rev::GFP*, *DR5rev::3XVENUS-N7*, *R2D2*, *PIN2-GFP*, *PIN3-GFP*, *BOR4-GFP*, *PEN3-GFP*, and *AUX1-YFP*, the 5-d-old, chambered coverslip (Lab-Tek)-mounted seedlings were placed on a Zeiss LSM700 microscope or Leica Stellaris 8 microscope. When using *DR5rev::GFP* reporter plants, GFP fluorescence intensities in the later root cap cells, located at 100 to 200 μm from the root tip, were analyzed to quantify lateral auxin distributions. For the analysis of PIN2-GFP polarity in root epidermal cells, atrichoblast cells were specifically used due to their greater length and clearer cellular PIN2 polarity compared to trichoblast cells (50). The GFP signal intensity was quantified by measurement of the mean gray value with Fiji software (https://imagej.net/Fiji). In planta FRAP experiments were performed using Leica confocal laser scanning microscopy Stellaris 8 (https://www.leica-microsystems.com/science-lab/life-science/step-by-step-guide-for-frap-experiments/).

### In Planta Hydrostimulation.

The 10-d-old *Arabidopsis* seedlings were transplanted into soil. In brief, seedlings were placed in rectangular soil-filled chambers. The chamber was composed of two transparent polystyrene plates (14 × 10 cm) and two blocks of water-absorbable cotton wool (2 × 14 cm), which were assembled by four clips. Two blocks of cotton wool were placed on the two sides of the chamber, parallel to the root system. To establish and maintain the experimental moisture gradient in the hydrostimulated chamber for a relatively long period of time, water was supplied to the cotton wool at one side of the chamber through an injection port every 5 d during the experiment. For the control without hydrostimulation, water was supplied to the cotton wool placed at both sides of the chamber. The chamber was placed at an angle of 10° deviated from the vertical to enhance root growth along the surface of the chamber. Plants were grown for 3 wk at 23 °C with a humidity of 60%. Images of root architecture were obtained by scanning the root system distributed on the bottom surface of the transparent plastic container with an image scanner (EPSON Perfection V370). The scanned images of roots were traced by visual discrimination of roots from the soil background using PowerPoint 2012. Then, the total root lengths between the two sides of the chamber were measured using ImageJ software (https://imagej.net/Fiji) to highlight their root system differences among different *Arabidopsis* lines after hydrostimulation.

## Supplementary Material

Appendix 01 (PDF)

Dataset S01 (DOCX)

Dataset S02 (XLSX)

## Data Availability

All study data are included in the article and/or supporting information.
