# Supplementary material for: Foraging for water by MIZ1-mediated antagonism between root gravitropism and hydrotropism
Source: Proc Natl Acad Sci U S A. 2025 May 15;122(20):e2427315122. doi: 10.1073/pnas.2427315122 (PMC12107133; doi:10.1073/pnas.2427315122)
Supplement: Supplementary file 1 — Appendix 01 (PDF) [file pnas.2427315122.sapp.pdf]

PNAS

# Supporting Information for
Foraging for water by MIZ1-mediated antagonism between root gravitropism and hydrotropism

Yuzhou Zhang, Zhulatai Bao, Adrijana Smoljan, Yifan Liu, Huihui Wang, Jiří Friml

Jiří Friml, Yuzhou Zhang
Email: jiri.friml@ist.ac.at, yuzhou.zhang@nwafu.edu.cn

**This PDF file includes:**

Figures S1 to S13
Legends for Datasets S1 to S2

## Supporting Information Text

**Subhead.** Type or paste text here. This should be additional explanatory text such as an extended technical description of results, full details of mathematical models, etc. Supporting information text for Brief Reports is limited to extended methods only.

## Heading

**Subhead.** Type or paste text here. You may break this section up into subheads as needed (e.g., one section on “Materials” and one on “Methods”).

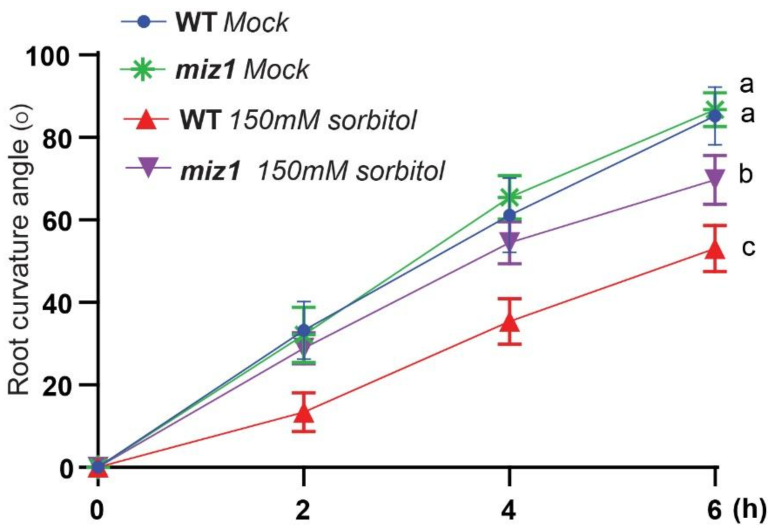


**Fig. S1. The root gravitropism of WT and *miz1* mutant with no treatment (Mock) or 150 mM sorbitol treatment.** Gravitropic curvatures were measured at 0, 2, 4, and 6 hours post-gravistimulation. Error bars represent s.e. from three biological replicates. n ≥ 10 roots for each replicate. Significant differences were determined by two-way ANOVA followed by Tukey's multiple comparisons test, with a significance level of $P < 0.001$.

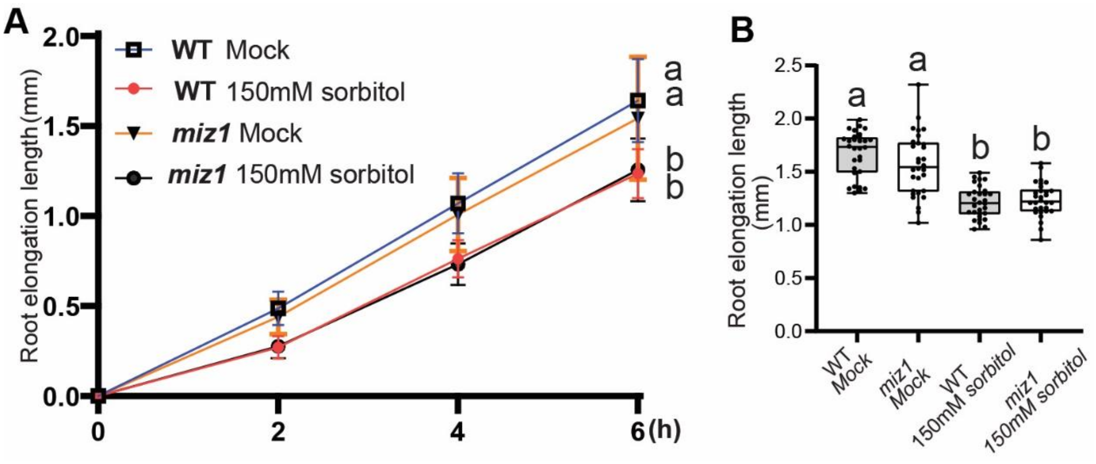


**Fig. S2. The comparison of root elongation rate between *miz1* and WT roots. (A)** Root elongation was measured in WT and *miz1* plants at 0, 2, 4, and 6 hours after the initiation of gravitropic stimulation. The measurements were taken under two conditions: no treatment (Mock) or 150 mM sorbitol treatment. Error bars represent s.e. from three biological replicates. $n \geq 10$ roots for each replicate. **(B)** Comparison of root elongation length between WT and *miz1* without or with 150 mM sorbitol treatment after 6 hours of gravistimulation. Each circle represents the measurement of an individual root. Boxplots span the first to the third quartiles of the data. Whiskers indicate minimum and maximum values. A line in the box represents the mean. Significant differences were determined by one-way ANOVA (B) or two-way ANOVA (A) followed by Tukey's multiple comparisons test, with a significance level of $P < 0.001$.

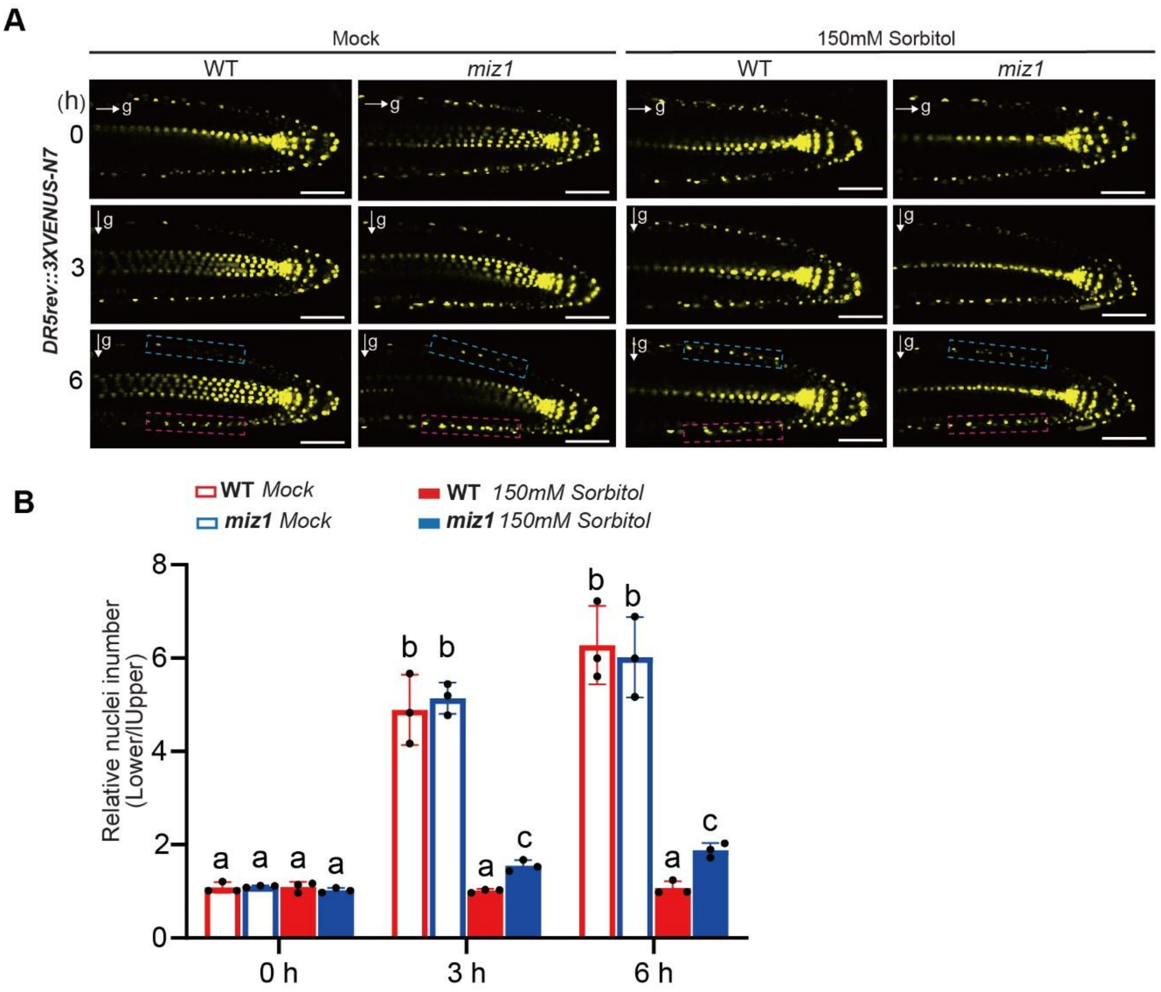


**Fig. S3. Analysis of the effect of MIZ1 on auxin asymmetry in *Arabidopsis* roots under osmotic stress. (A)** Auxin redistribution was examined in 7-day-old WT and *miz1* mutant plants under no treatment (Mock) and 150 mM sorbitol treatment. The auxin-responsive reporter *DR5rev::3xVENUS-N7* was used to visualize auxin redistribution. Images were taken at 0, 3, and 6 hours after the initiation of gravistimulation. Scale bars, 50 µm. **(B)** Quantitative analysis of the ratio of *DR5rev::3xVENUS-N7* expressing nuclei in epidermal cells between upper and lower sides of gravistimulated roots shown in (A). Error bars represent s.e. from three biological replicates, with each replicate consisting of at least 5 roots (n ≥ 5). Significant differences were determined by one-way ANOVA followed by Tukey’s multiple comparisons test, with a significance level of $P < 0.001$.

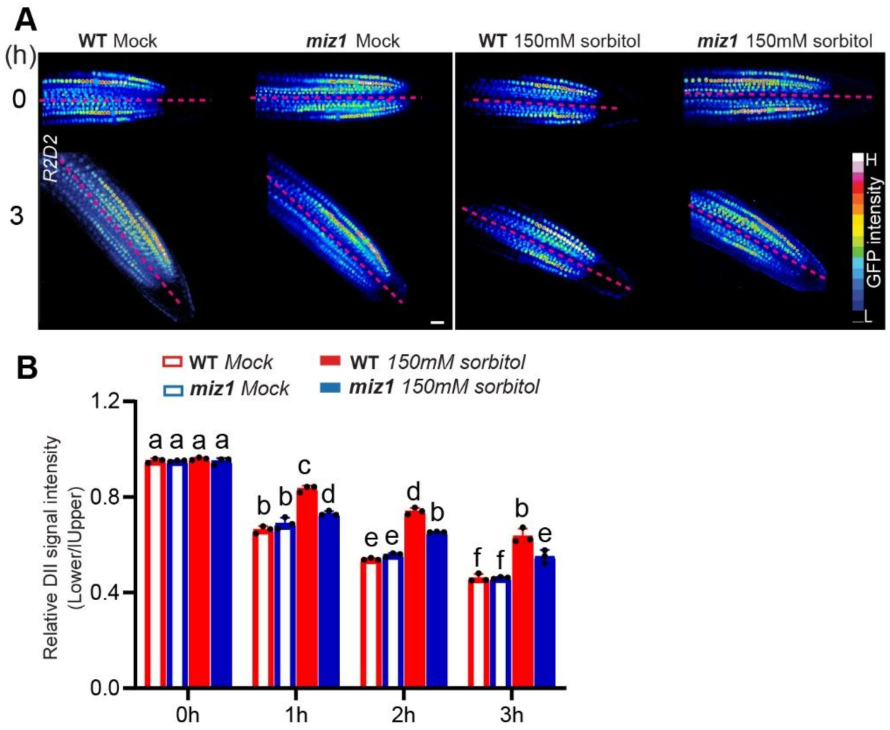


**Fig. S4. MIZ1 enhances the inhibitory effect of low environmental Wp (sorbitol treatment) on auxin asymmetry in *Arabidopsis* root, as indicated by the auxin sensor *R2D2*. (A)** Auxin redistribution was observed in 7-d-old WT and *miz1* mutant with no treatment (Mock) or 150 mM sorbitol treatment, following 3 hours of gravitropic stimulation. The Aux/IAA-based auxin signaling sensor DII-VENUS (yellow fluorescent protein) channel images are presented as LUT images, with the intensity scale displayed on the right (H, high; L, Low). Scale bars, 20 μm. **(B)** The ratio of DII-VENUS intensity between the lower and upper parts of the roots was measured at 0, 1, 2, and 3 hours after gravistimulation. Error bars represent s.e. from three biological replicates, with each replicate consisting of at least 5 roots ($n \geq 5$). Significant differences were determined by one-way ANOVA followed by Tukey's multiple comparisons test, with a significance level of $P < 0.001$.

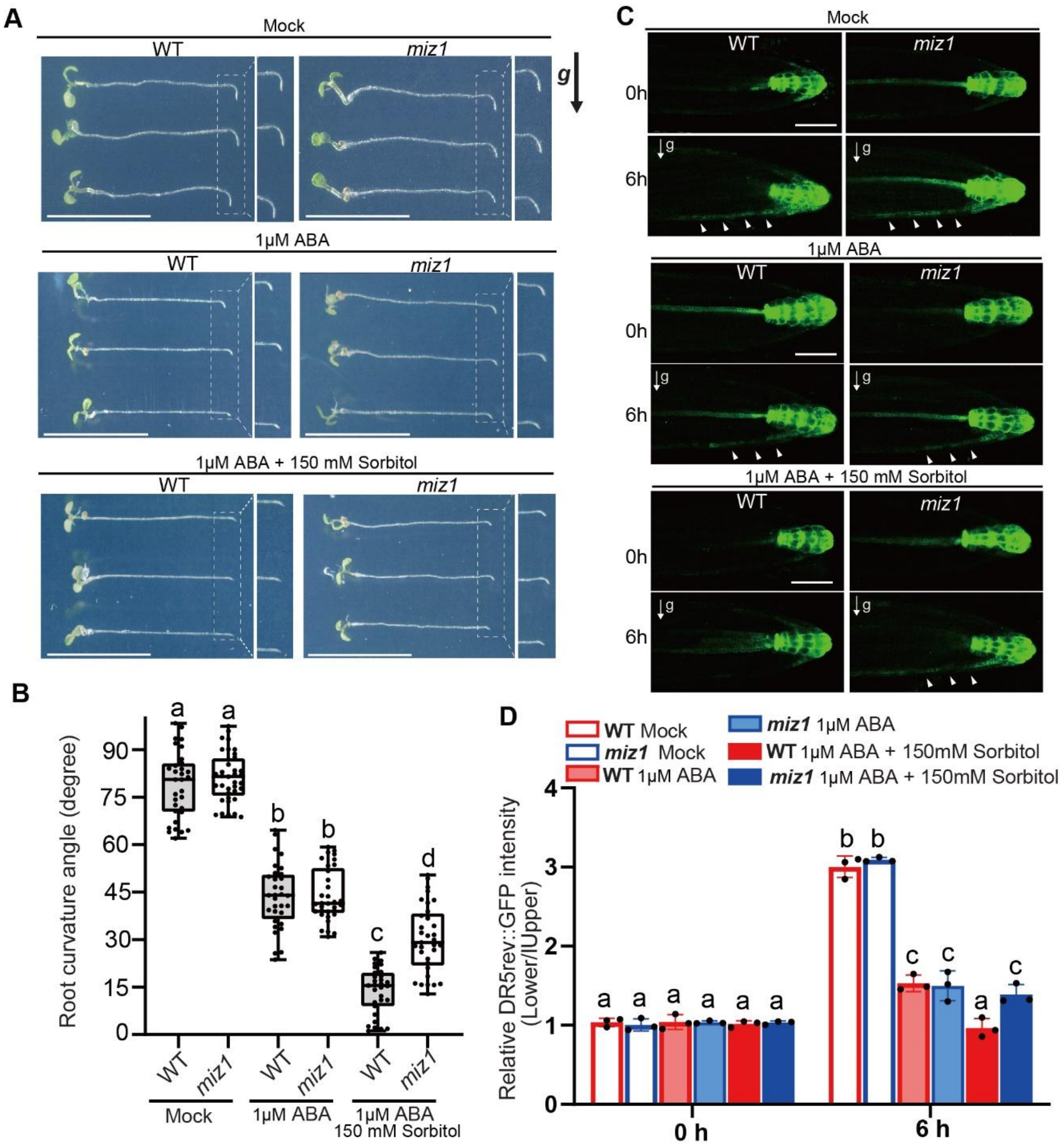


**Fig. S5. ABA signaling does not play a role in the MIZ1-mediated regulation of dynamic auxin asymmetry formation and root gravitropism under osmotic stress. (A)** Phenotypic analysis of root gravitropism in 7-day-old *Arabidopsis* WT and *miz1* mutant plants under different treatments: no treatment (Mock), 1 μM ABA treatment, or 150 mM sorbitol plus 1 μM ABA treatment. Roots were gravistimulated for 6 hours following a 90° reorientation. Scale bars, 1 cm. **(B)** Quantification of root bending curvature from (A) for each group. Data are derived from three biological replicates for each group, with at least 10 roots per replicate (n ≥ 10). Boxplots span the first to the third quartiles of the data. Whiskers indicate minimum and maximum values. A line within each box represents the mean. **(C)** Auxin redistribution in roots of WT and *miz1* mutant under no treatment (Mock), 1 μM ABA treatment, or 150 mM sorbitol plus 1 μM ABA treatment after 6 hours of gravistimulation, as indicated by the synthetic auxin-responsive reporter *DR5rev::GFP*. Scale bars, 50 μm. **(D)** Statistical analysis of *DR5rev::GFP* intensity ratio between the lower and upper lateral root flanks from (C). Error bars represent s.e. from three biological replicates, with each replicate consisting of at least 5 roots (n ≥ 5). Significant differences were

determined by one-way ANOVA followed by Tukey’s multiple comparisons test, with a significance level of $P < 0.001$.

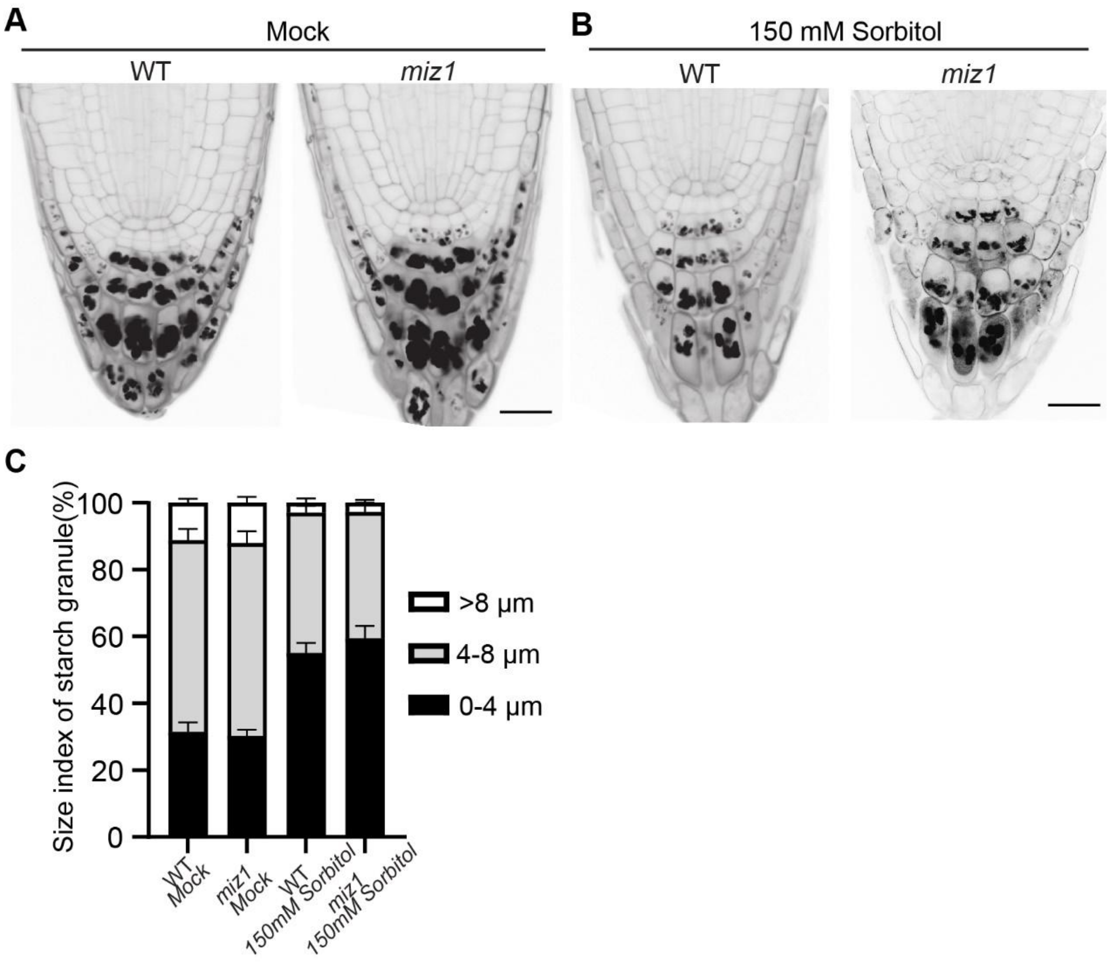


**Fig. S6. The decrease in amyloplasts induced by osmotic stress in the *Arabidopsis* root apex does not depend on the MIZ1-mediated signaling. (A-B)** Observation of amyloplasts in the root apex of 7-day-old WT and *miz1* mutant without treatment (A) or with 3 hours of 150 mM sorbitol treatment (B). Scale bars, 20 μm. **(C)** Quantification of amyloplast size shown in (A) and (B). Error bars represent s.e. from three biological replicates. No fewer than 20 cells from 5 roots were analyzed for each replicate.

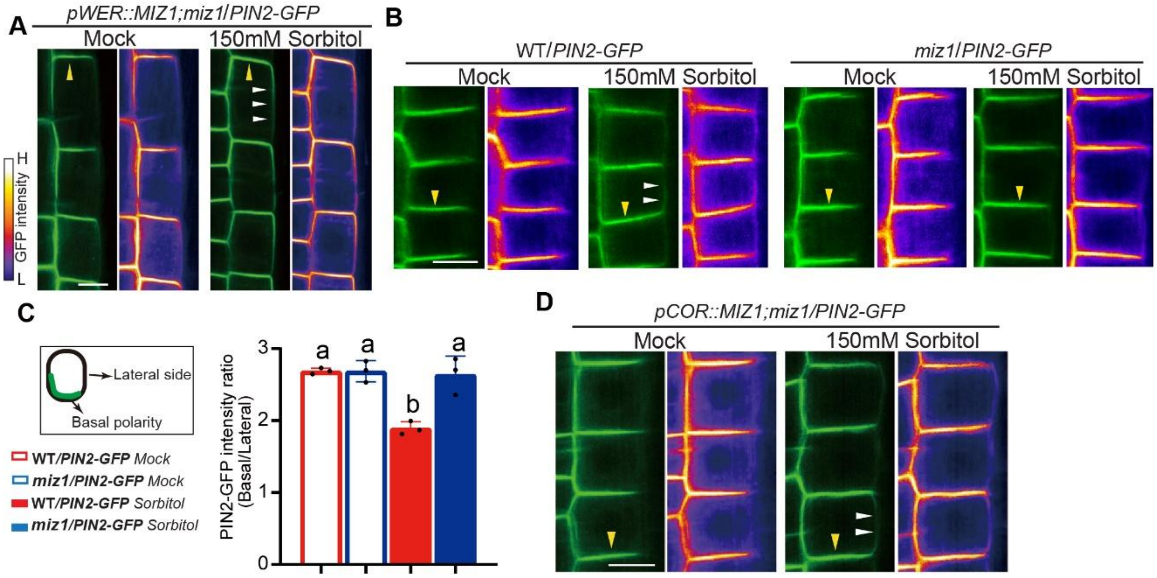


**Fig. S7. MIZ1 modulates the polarity of PIN2 in both root epidermal and cortex cells under osmotic stress. (A)** The polarity dynamics of PIN2-GFP at the PM of epidermal cells (specifically atrichoblasts) in the *pWER::MIZ1;miz1/PIN2-GFP* line were assessed in response to 150 mM sorbitol treatment. **(B)** Localization of PIN2-GFP at the PM of root cortex cells in WT and *miz1* mutant plants, both without treatment (Mock) and after treatment with 150 mM sorbitol. **(C)** Quantitative analysis of the ratio of PIN2-GFP signal intensity between the basal and lateral sides of cortex cells shown in (B). Error bars represent s.e. from three biological replicates. No fewer than 20 cortex cells from 5 roots were analyzed for each replicate. Significant differences were determined by one-way ANOVA followed by Tukey's multiple comparisons test, with a significance level of $P < 0.001$. **(D)** The polarity dynamics of PIN2-GFP at the PM of cortex cells in the *pCOR::MIZ1;miz1/PIN2-GFP* line were assessed in response to 150 mM sorbitol treatment. Yellow arrowheads indicate the apical/basal polarity of PIN2-GFP in root epidermal/cortex cells, while white arrows indicate the lateral PIN2-GFP signal in root epidermal/cortex cells. Scale bars, 10 μm. GFP channel images are also presented as LUT images, with the intensity scale shown to the right (H, high; L, Low).

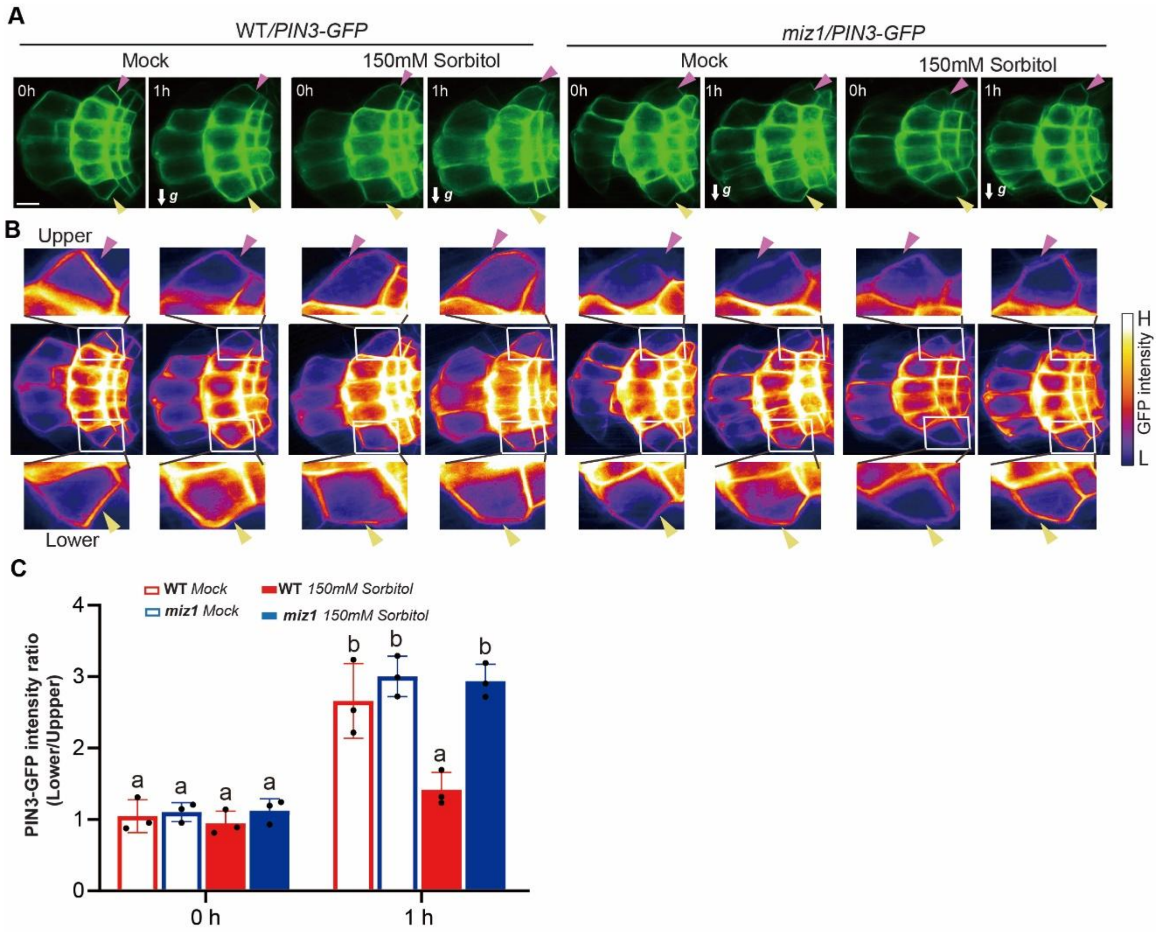


**Fig. S8. MIZ1 reduces gravity-induced PIN3 repolarization under osmotic stress, facilitating root hydrotropic bending. (A-B)** Visualization of gravity-induced PIN3 repolarization at the PM of columella cells in WT and *miz1* mutant plants under control condition (Mock) and with 150 mM sorbitol treatment. Scale bars, 10 μm. Yellow arrowheads indicate PIN3-GFP intensity on the lower side of columella cells, while magenta arrowheads show the intensity of the PIN3-GFP on the upper side. GFP channel images are also presented as LUT images (B), with the intensity scale shown on the right (H, high; L, Low). Additionally, zoomed-in views of the lower and upper sides of columella cells in the root apex are provided to highlight the asymmetric PIN3 expression between the upper and lower sides of the root. **(C)** Quantitative analysis of PIN3-GFP repolarization in (A-B). The ratios of PIN3-GFP signal intensity between the upper side (magenta arrowheads) and the lower side (yellow arrowheads) of columella cells were measured before and after 1 hour of gravistimulation. Error bars represent s.e. from three biological replicates. No

fewer than 10 roots were analyzed per replicate. Significant differences were determined by one-way ANOVA followed by Tukey's multiple comparisons test, with a significance level of P < 0.001.

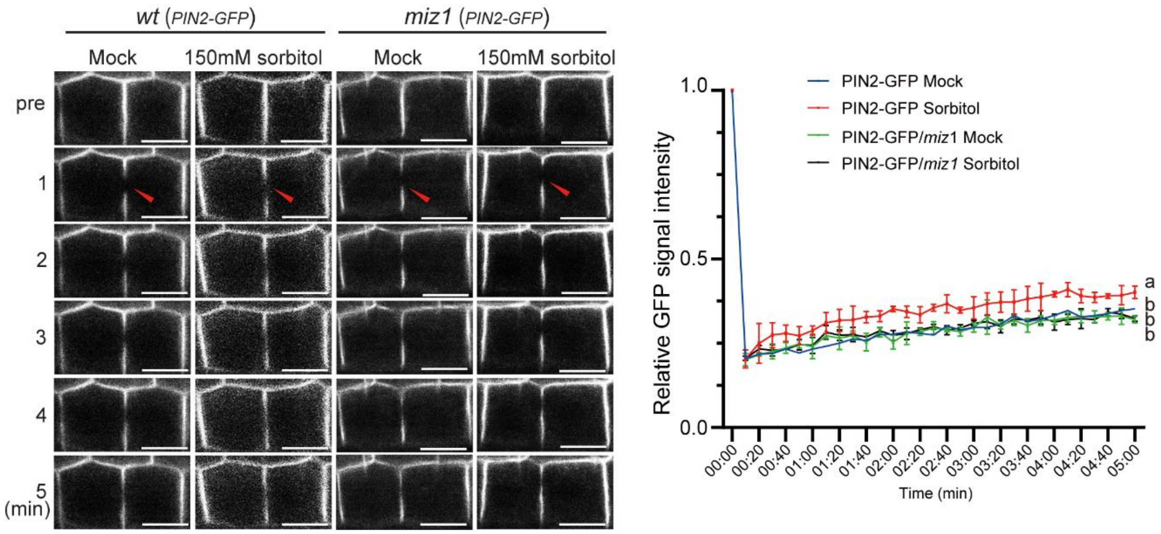


**Fig. S9. MIZ1 promotes osmotic stress-induced lateral diffusion of PIN2 in the PM of root cortex cells. (A)** FRAP dynamics of PIN2-GFP in root cortex cells of WT and *miz1* mutant with no treatment or 150 mM sorbitol treatment. Scale bars, 10 μm. **(B)** Quantitative analysis of the fluorescence recovery rate of PIN2-GFP after photobleaching in (A). Error bars represent s.e. from three biological replicates. No fewer than 20 cells from 5 roots were analyzed for each replicate. Significant differences were determined by two-way ANOVA followed by Tukey's multiple comparisons test, with a significance level of P < 0.001.

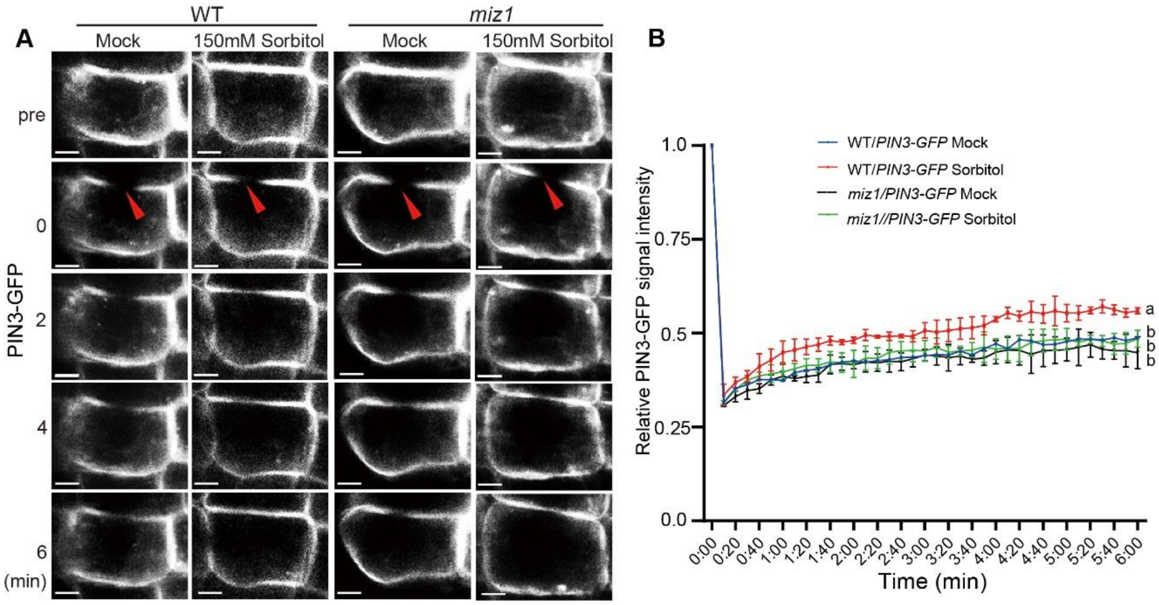


**Fig. S10. MIZ1 enhances osmotic stress-induced lateral diffusion of PIN3 in the PM of root columella cells. (A)** FRAP analysis of PIN3-GFP in root columella cells of WT and *miz1* mutant with no treatment (Mock) and 150 mM sorbitol treatment. The FRAP dynamics indicate the mobility of PIN3-GFP in the PM. Scale bars, 10 μm. **(B)** Quantitative analysis of the fluorescence recovery rate of PIN3-GFP after photobleaching shown in (A). The recovery rate is used to assess the lateral diffusion of PIN3-GFP. Error bars represent s.e. from three biological replicates. No fewer than 10 roots were analyzed per replicate. Significant differences were determined by two-way ANOVA followed by Tukey's multiple comparisons test, with a significance level of $P < 0.001$.

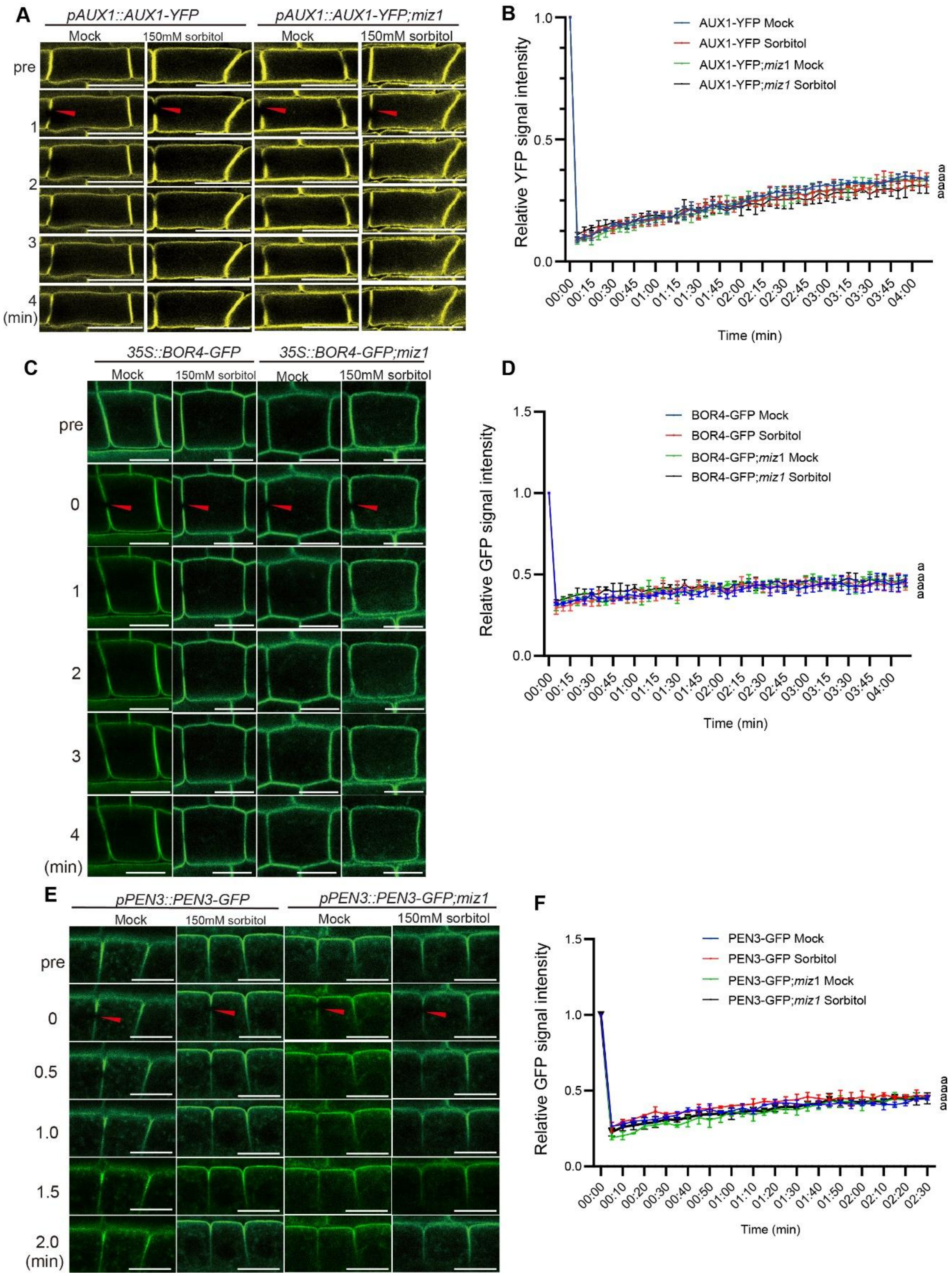


**Fig. S11. MIZ1 does not significantly enhance the lateral diffusion rates of PM proteins AUX1, BOR4, and PEN3 in the PM of root cells under osmotic stress. (A, C, E)** FRAP analysis of AUX1-YFP, BOR4-GFP, and PEN3-GFP in root epidermal cells of WT and *miz1* mutant. The analysis was conducted under two conditions: no treatment (Mock) and 150 mM

sorbitol treatment. The FRAP dynamics indicate the mobility of AUX1-YFP, BOR4-GFP, and PEN3-GFP in the PM. Scale bars, 10 μm. **(B, D, F)** Quantitative analysis of the fluorescence recovery rate of AUX1-YFP (A), BOR4-GFP (C), and PEN3-GFP (E) after photobleaching. The recovery rate is used to assess the lateral diffusion of AUX1-YFP, BOR4-GFP, and PEN3-GFP. Error bars represent s.e. from three biological replicates. No fewer than 10 roots were analyzed per replicate. Significant differences were determined by two-way ANOVA followed by Tukey's multiple comparisons test, with a significance level of $P < 0.001$.

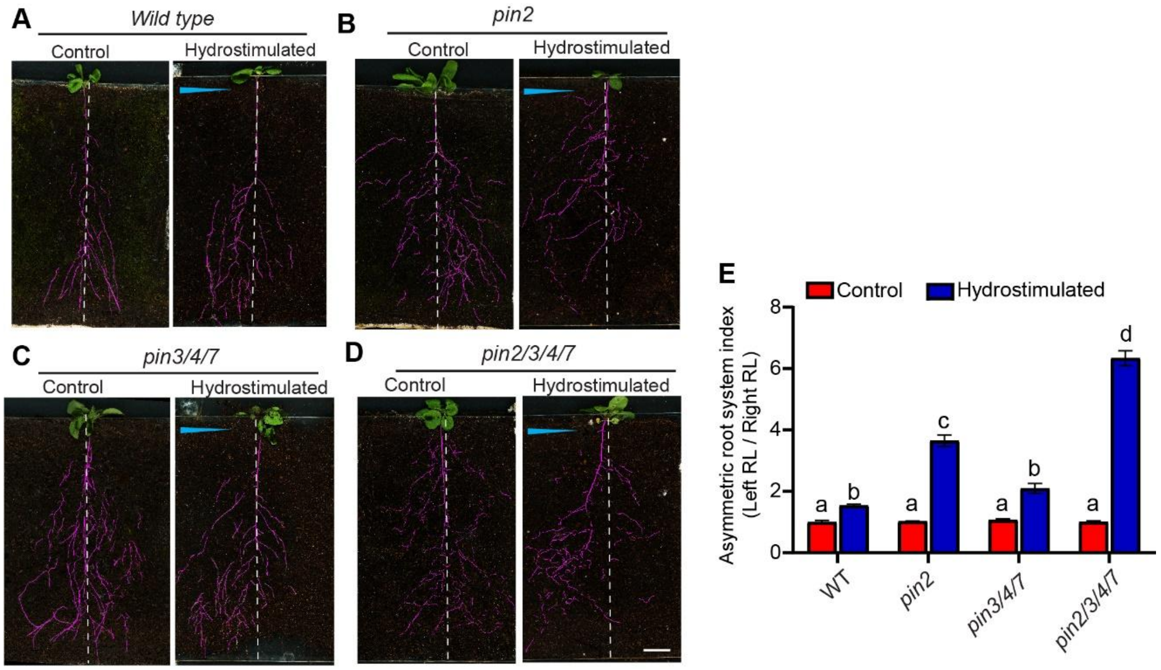


**Fig. S12. Inactivation of the auxin-efflux transporter PIN function enhances the hydrotropism of the *Arabidopsis* root system to facilitate drought avoidance. (A-D)** Visualization of the root system in the absence of hydrostimulation (Control) and in the presence of hydrostimulation. The root systems of different genotypes are shown: (A) WT, (B) *pin2* mutant, (C) *pin3/4/7* mutant, and (D) *pin2/3/4/7* mutant. Scale bars, 2 cm. **(E)** Quantitative analysis of the asymmetric index of the root system in (A-D) by calculating the ratio between the left total root length (TRL) and right TRL. Error bars represent s.e. from three biological replicates, with each replicate including three plants. Significant differences were determined by one-way ANOVA followed by Tukey's multiple comparisons test, with a significance level of $P < 0.001$.

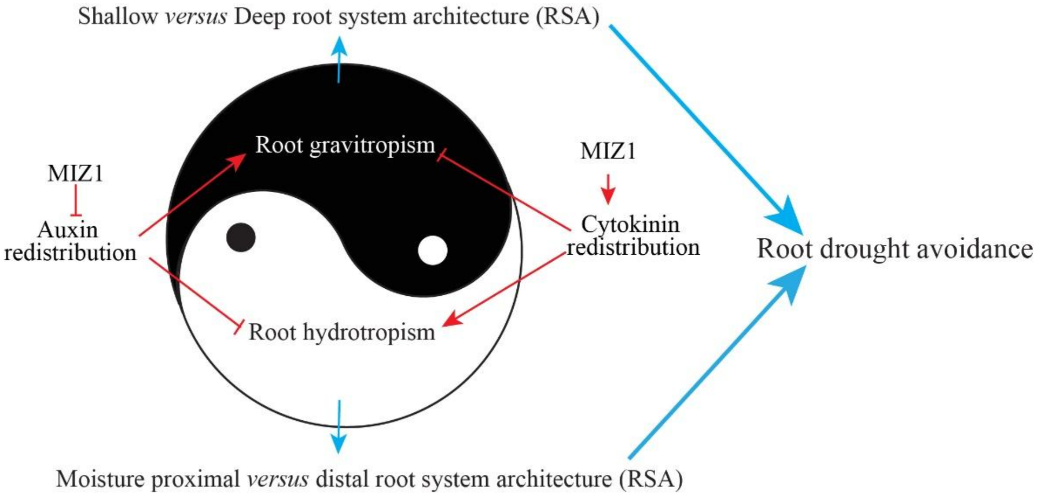


**Fig. S13. Proposed model of the antagonistic interplay between cytokinin and auxin in regulating root gravitropism-hydrotropism tradeoffs to shape root system architecture (RSA) for drought avoidance.** Our work, combined with previous reports, suggests that MIZ1 positively regulates cytokinin redistribution (Ref. 20), while negatively regulating auxin redistribution in roots to promote *Arabidopsis* root hydrotropism. On the other hand, cytokinin redistribution impedes *Arabidopsis* root gravitropism (Ref. 41), while auxin redistribution promotes root gravitropism. These opposing effects of cytokinin and auxin play a crucial role in shaping the root system architecture in both vertical (shallow vs. deep) and water potential gradient (moisture proximal vs. distal) orientations, enabling plant roots to avoid drought.

**Dataset S1.** List of primers used in this study.

**Dataset S2.** Original data used for statistical analysis.
