# Supplementary material for: Foraging for water by MIZ1-mediated antagonism between root gravitropism and hydrotropism
Source: Proc Natl Acad Sci U S A. 2025 May 15;122(20):e2427315122. doi: 10.1073/pnas.2427315122 (PMC12107133; doi:10.1073/pnas.2427315122)
Supplement: Supplementary file 2 — Dataset S01 (DOCX) [file pnas.2427315122.sd01.docx]

| Gene | Primer sequences |
| --- | --- |
| Primers used for vector construction | |
| *WER* promoter F | 5’- CGGTACCCGGGGATCTTCCGACCTTAAAGCTCCTACAAAA-3’ |
| *WER* promoter R | 5’- CGACTCTAGAGGATC TCTTTTTGTTTCTTTGAATGATAGA-3’ |
| *COR* promoter F | 5’-TATAGAGCGAATTGGCGTGCGGTCAAATAACAGAAG-3’ |
| *COR* promoter R | 5’-CTTGAGCTCGGTCGAGGTTTTGGCTAATGTGATTGTG-3’ |
| *MIZ1* CDS F | 5’-GGGGACAAGTTTGTACAAAAAAGCAGGCTTCATGGTGCCATACCAAGAACTCACTCTT-3’ |
| *MIZ1* CDS R | 5’-GGGGACCACTTTGTACAAGAAAGCTGGGTCTTATATTCTAAGAAGAAAAATACTGAG-3’ |
| Primers used for *miz1* mutant genotyping | |
| LP-MIZ1 | 5’- GGAGATGCTCCTATGTCCCTC-3’ |
| RP-MIZ1 | 5’- GTAAAAGCAAATTCGCATTGC-3’ |
| LBb1.3 | 5’- ATTTTGCCGATTTCGGAAC-3’ |
